# Assessment of community health workforce governance in federal Nepal

**DOI:** 10.1093/heapol/czaf088

**Published:** 2026-06-29

**Authors:** Roosa Sofia Tikkanen, Sushmita Pokhrel, Usha Ghimire, Biraj Neupane, Svea Closser, Bernadette Nirmal Kumar

**Affiliations:** Department of Sociology and Political Science, Faculty of Social and Educational Sciences, Norwegian University of Science and Technology, Edvard Bulls veg 1, 7491 Trondheim, Norway; Department of Sociology and Political Science, Faculty of Social and Educational Sciences, Norwegian University of Science and Technology, Edvard Bulls veg 1, 7491 Trondheim, Norway; Institute of Applied Health Sciences, School of Medicine, Medical Sciences and Nutrition, 3Rd Floor Health Sciences Building, University of Aberdeen, Foresterhill, Aberdeen AB25 2ZD, United Kingdom; Community Health Division, Kathmandu University School of Medical Sciences, Dhulikhel Hospital, Dhulikhel 45200, Kavrepalanchowk, Nepal; School of Information Sciences, University of Illinois Urbana-Champaign, 614 E. Daniel Street, Champaign, IL 61820, United States; Johns Hopkins Bloomberg School of Public Health, 615 N. Wolfe Street, Baltimore, MD 21205, United States; Norwegian Public Health Institute, Postboks 222 Skøyen, 0213 Oslo, Norway; Empower School of Health, 3rd Floor, Raheja Mall, Suite # 325, Sohna Road, Sector 47, Gurugram, Haryana 122018, India

**Keywords:** community health, governance, decentralization, health workforce, health workers, local government

## Abstract

How Community Health Worker (CHWs) programs are governed shapes their performance. CHW governance can be challenged by CHWs’ accountability to both governments and their communities, and the need to coordinate between multiple actors. Furthermore, little is known about how decentralization impacts CHW governance. We examined Nepal’s Female community health volunteers (FCHV), where a 2015 constitutional change to federalism—a major governance reform representing one form of decentralization—shifted the responsibility for community health governance to local municipalities. We assessed FCHV governance in the federal landscape and provide preliminary insights into how federalization has impacted FCHV governance in its early years. We identified opportunities and challenges brought by federalization for six actors: federal, provincial and municipal governments, international donors, FCHVs and their representative organizations (unions), and non-governmental organizations. Our qualitative case study comprised 26 semi-structured interviews and five focus groups conducted with FCHVs, health workers, federal and municipal government representatives, researchers, and FCHV union, donor and NGO representatives, across four districts in Bagmati Province, combined with analysis of 259 documents. We analysed these data using a theoretical framework of CHW governance adapted from seven existing frameworks on health workforce and community health governance. We show that FCHV governance was characterized by improved accountability, transparency, participation, and responsiveness to local needs, to some extent. Decentralization remained incomplete, with governance challenged by limited budgetary and administrative decision-space at local levels, in part because of continued central-level control and limited revenue-raising capacity, coordination, and enforcement challenges, as well as lack of role clarity. While donors and NGOs participate in decision-making, FCHVs and their representative organizations are not consistently consulted. The results indicate that strengthening FCHV governance in federal Nepal will require capacity-building of municipalities while loosening the federal grip.

Key Messages

- How community health workers (CHWs) are governed shapes their performance, but few analyses of CHW governance exist. We examined how the governance of Nepal’s main CHW cadre, the female community health volunteers (FCHVs) has been impacted by the country’s transition towards federalism in 2015
- Under federalism, certain aspects of FCHV governance such as accountability, transparency, participation, and responsiveness to local needs have been strengthened, while limited budgetary and administrative decision-space at local levels (municipalities), as well as enforcement, remain as challenges
- While donors and NGOs have participated in decision-making around community health, FCHVs and their representative organizations (unions) are not consistently included
- CHW governance in federalizing contexts requires attention towards clear role delineation across actors and government tiers and enabling institutional capacity at local levels

## Introduction

Community health workers (CHWs) are central to primary health care delivery, which is a cornerstone of universal health coverage. How CHW programs are governed impacts how they are managed, resourced, and ultimately, how programs perform (Lewin et al. 2021). Yet, CHW governance is an understudied area (Scott et al. 2018, Agarwal et al. 2019, Blanchard et al. 2019), particularly in decentralized contexts where authority over public functions is transferred from central to local governments (Scott et al. 2018, Boex et al. 2022).

Governance is frequently conceptualized as an essential health system ‘function’ or ‘building block’ (WHO 2007, Kruk et al. 2018). Governance entails ‘the exercise of political, economic and administrative authority’ (UNDP 1997) involving interactions between ‘actors’— policymakers, private actors, and civil society —and ‘structures’— laws, policies, resources, and beliefs (Kooiman et al. 2008). Decentralization is a political reform that in theory improves governance by enhancing ‘responsivity’ to local needs, ‘efficiency’ of resource use, ‘participation’ of marginalized groups, as well as ‘accountability’ and ‘transparency’ of local representatives to their constituents. Yet, real-world evidence on decentralization impacts tend to be mixed (Abimbola et al. 2019): Negative impacts, such as increased corruption and patronage, lack of local autonomy, and poor coordination, are often observed early on. Over time, positive impacts, including increased mutual accountability and community participation in decision-making (Sapkota et al. 2023), may emerge. Key to the success of decentralization is the degree of ‘decision space’ granted to subnational governments over functions and the exercise of power (Bossert 1998), adequate institutional capacities, including leadership and management at local levels, and mutually accountable relationships between government tiers (Bossert and Mitchell 2011, Abimbola et al. 2019).

Health workforce governance is typically characterized as either ‘process-oriented’, namely, how rules and responsibilities are distributed across actors, ‘outcomes-oriented’, i.e. whether policies are aligned with health system objectives, or a mix of both (Lim and Lin 2021). Given that CHWs are a subset of the health workforce, ‘CHW governance’ can equally be considered a subset of health workforce governance. CHW governance is theoretically inherent with several challenges. Since CHWs serve as bridges between communities and health systems, they are dually-accountable to both (Schaaf et al. 2020) while typically being poorly integrated into health systems (Tulenko et al. 2013). CHW governance requires coordinating across actors, including governments, international donors—who fund most CHW programs in low- and middle-income countries (LMICs; Gichaga et al. 2021)—non-governmental organizations (NGOs), and CHWs' representative organizations (Schaaf et al. 2018, Trafford et al. 2018, Sriram et al. 2023). In decentralized settings, further coordination is needed across national and subnational governments (Schneider and Nxumalo 2017, Perry et al. 2021a).

Few studies exist on CHW governance in decentralized settings. In South Africa, decentralization enabled adapting CHW programs locally and increased participatory planning but created challenges around role definitions and local fiscal autonomy (Schneider and Nxumalo 2017). In the Philippines, decentralization resulted in variation in local capacity to train and remunerate CHWs (Dodd et al. 2021). In Pakistan, devolution (one form of decentralization) led to role confusion across government tiers (Kundi 2012).

Federalism is a form of decentralization characterized by self-rule and shared rule, wherein the legislative powers of local government are constitutionally protected (Elazar 1991, Tremblay 2018). Nepal is one of the world’s youngest federations, with federalism introduced through the 2015 constitution. This paper assessed the governance of Female Community Health Volunteers (FCHVs)—the country’s largest CHW cadre—under federalism. A secondary goal was to assess how governance structures changed under federalism. FCHVs make for an interesting case as they have been hailed as a success because of their low turnover rates and contributions towards reducing maternal and child deaths (Panday et al. 2017, Perry 2021b). We aim to contribute towards strengthening FCHV governance and facilitating progress towards two constitutional goals: universal access to basic health care and ‘good governance’.

### Nepal’s move to federalization and health sector governance

Nepal’s 2015 Constitution introduced ‘cooperative federalism’, wherein the federal, seven newly-created provincial, and 753 newly-created municipalities, or *palikas*, are expected to cooperate in policymaking and service delivery (Thaneshwar 2023). Nepal started practicing federalism in 2018, following the 2017 local elections—the first in two decades (MoHP 2022). Early evidence suggests that federalism has increased self-rule and shared rule (OECD and ADB 2023), but top-down approaches persist in political, administrative, and fiscal processes (Thaneshwar 2023).

Prior fiscal decentralization reforms through the 1999 Local Self Governance Act made ‘districts’ the basic unit of governance and responsible for primary health care. The goals of this reform were not achieved in large part because of the 1996–2006 civil war (Asia Foundation 2017). Despite some added ‘decision space’ at local governments towards revenue generation and planning, they had limited-to-no role in policy formulation, resource allocation, implementation, or monitoring (Ministry of Local Development 2002, Regmi et al. 2010, Bhandari et al. 2020).

Under the 2015 constitution, health is a shared responsibility across government tiers, but primary health care is the exclusive responsibility of *palikas* (Government of Nepal 2015), which have wide legislative, budgetary, and executive powers. The federal government is responsible for policy formulation, budgeting, and regulation; Provinces for basic hospitals. Research to date suggests federalism has allowed tailored local planning, increased *palika* health budgets and their ability to flexibly hire health workers, and improved access to essential medicines. Meanwhile, confusion around role clarity across the three government tiers, limited decision-making power and inadequate *palika-*level human resources and continued central control of policy, planning, and financing continue to constrain achieving federalism as constitutionally envisioned (Regmi 2018, Bhandari et al. 2020, Adhikari et al. 2022, Bhattarai et al. 2022, Nepal Federal Health System Project 2022, Sapkota et al. 2022, Brennan and Abimbola 2023, Chen et al. 2023, Wasti et al. 2023).

### Nepal’s FCHVs

The Ministry of Health and Population (MoHP) introduced FCHVs in 1988. FCHVs are to be selected by women’s groups known as Healthy Mothers’ Groups (HMGs) according to government criteria, although in reality, selection practices vary (Tikkanen et al. 2024). FCHVs provide basic preventive and promotive services and referrals to health facilities, where they receive training and supervision. Program goals were originally focused on maternal and child health but gradually expanded to include communicable diseases. Although volunteers, they receive work-related incentives such as uniform and transport allowances and a one-time retirement payment upon turning 60 years. They are also entitled to loans from a locally-run credit savings ‘FCHV Fund’.

No prior studies have assessed FCHV governance explicitly. Pre-federal studies including the latest national evaluation from 2014 (MoHP 2015) indicated governance challenges such as poor coordination across donor-funded programs; inconsistent provision of FCHV incentives; supervision and training gaps; supply chain issues; and monitoring gaps (Schwarz et al. 2014, Khatri et al. 2017, Panday et al. 2017, Adhikari et al. 2022). FCHV dissatisfaction with their incentives and lack of participation in decision-making has contributed to demands for greater recognition (Panday et al. 2017, 2024) and the formation of representative organizations—which we here call ‘unions’, given their affiliation with trade union federations (Glenton et al. 2010, PSI 2018, Tikkanen et al. 2024). Under federalism, management, planning, and supply issues persist (Joshi et al. 2024), and the past decade has seen questions around the appropriateness of FCHVs—around 17% of whom were illiterate as of 2014—in meeting the rising burden of non-communicable diseases (NCDs) (MoHP 2015, WHO 2018a, Tikkanen et al. 2024). Some have argued that federalization allows the opportunity for *palikas* to strengthen FCHVs or introduce more ‘professional’ cadres (Nepal et al. 2020).

## Methods

### Analytical framework

We constructed an analytical framework of CHW governance using Siddiqi et al’s. (2009) health system governance framework as a starting point, identified from a review on CHW governance (Lewin et al. 2021). It identified ten governance ‘principles’: ‘strategic vision, participation and consensus orientation, rule of law, transparency, responsiveness, equity and inclusiveness, effectiveness and efficiency, accountability, intelligence and information’, and ‘ethics’, representing governance ‘values’ from the UNDP’s (1997) ‘good governance’ framework, and governance ‘functions’ such as policy, enforcement, and monitoring and evaluation. The framework offered 61 guiding questions for assessing national and sub-national governance in LMICs, and has been previously applied to Nepal (Petersen et al. 2017, Upadhaya et al. 2017).

To ensure relevance to CHW governance, we compared Siddiqui’s framework to six frameworks on health workforce or community health governance (Dieleman et al. 2011, Kaplan et al. 2013, Chen et al. 2021, Lim and Lin 2021, Sonderegger et al. 2021, Martineau et al. 2022), identifying dimensions in common across frameworks and their definitions. This resulted in removing ‘ethics’ given that it was not commonly included and some of its components covered in other dimensions, and adding ‘institutional capacity’ encompassing budgeting, leadership and coordination capacity (Lim and Lin 2021, Sonderegger et al. 2021, Martineau et al. 2022). We merged these into one analytical category rather than treating implementation as one of Siddiqui’s three analytical ‘levels’ (national, policy formulation, implementation), because these levels conflate government tiers with government functions: e.g. in federal Nepal, policy formulation is the responsibility of all three government tiers rather than exclusively of the national government. We also merged ‘accountability’ and ‘transparency’ because the concepts are interrelated. Our final analytical questions are show in Table 1; further details are in the Supplementary File.

**Table 1.** Analytical CHW governance framework.

Dimension	Sub-dimension	Operationalisation for assessment: research questions
**Inputs:** Institutional structures such as formal and informal rules and division of responsibilities	Strategic vision	Are the expected roles and responsibilities (incl. ownership) of governments and other stakeholders clarified in strategic plans? (14,7) Are strategic goals and priorities for the CHW program formulated in policies/plans? (1–7)
	Participation and consensus-orientation	Are international donors (development partners), civil society actors, health worker associations (unions), private actors and other stakeholders involved with or represented in decision-making structures and processes? (1,2,3,4,5,6,7) How are differing interests across stakeholders negotiated and reconciled in decision-making? (1,2,3,6,7)
**Processes**: Implementation and execution of rules and responsibilities, incl. administrative procedures and oversight	Institutional capacity and design	Do institutions and individuals have the necessary capacities to implement goals specified in the strategic vision (e.g. financial, management, leadership, human resources, coordination)? (3,4,5,6^a^,7)
	Accountability & Transparency	How is health system accountability to FCHVs ensured (an enabling environment in terms of supervision, training)? (14,7) How is FCHV accountability to the health system and community ensured (e.g. performance, voicing community concerns)? (3,4,5,7) How transparent are decision-making processes? (1,4,5,7)
	Reporting & Information systems	What information generation and data reporting mechanisms exist toward monitoring and evaluation? (1,2,3,4,5,6,7) Is this data made available and utilized in evidence-based planning by decision-makers? (14,5)
	Rule of law & Enforcement	How are policies, laws, and regulations enforced (e.g. penalties for non-compliance)? (1,2,3,5)
**Outputs**: Positive aims that health system governance should generate	Responsiveness	How is responsiveness to local health needs ensured (disease burden, competencies)? (1,2,4,5) How is responsiveness to CHW needs ensured (motivation, satisfaction)? (5)
	Equity & Equality	What policies are in place to ensure CHWs reach underserved/marginalized areas and populations? (1,2) How is fair treatment of CHWs ensured (e.g. compensation differentials, by ethnicity)? (3,4)
	Efficacy and efficiency	Are goals specified in CHW strategic plans achieved efficiently (i.e. without duplication or waste)? (1,2,3,4,5,7)?

Sources: (1) Siddiqi et al. 2009, (2) Lim and Lin 2021, (3) Dieleman et al. 2011, (4) Kaplan et al. 2013, (5) Sonderegger et al. 2021, (6) Martineau et al. 2022, (7) Chen et al. 2021.

CHW, Community health worker; FCHV, Female community health volunteer.

^a^ Martineau et al. (2022) refer to ‘capacity of human resources unit staff’ as ‘a professionalized body of HRH (human resources for health) scientists and planners as well as policy-makers who understand and can support HRH at a strategic level’. They also coin the term ‘health workforce literacy’, referring to the ability to think about terms and conditions of employment, productivity, performance and labor rights. We treat these concepts within the concept of ‘management and leadership capacity’ of individual leaders.

We organized governance dimensions into: ‘inputs, processes, and outputs’ (Baez-Camargo and Jacobs 2011), a conceptual trinity commonly used in health systems assessments (e.g. Donabedian 2005, Kruk et al. 2018, Sonderegger et al. 2021, Leslie et al. 2023). Because our governance definition relied on the interplay between ‘structures’ and ‘actors’, we identified opportunities and challenges presented by federalism for six actors: federal, provincial and *palika* governments, international donors, NGOs and FCHVs and their unions (Martineau et al. 2022).

### Data and analysis

Primary and secondary data were used to assess FCHV governance under federalism. Secondary data were the main data source for assessing governance structures before and after federalism.

#### Primary data

##### Study design and setting

Cross-sectional observational case study of four districts (Dolakha, Kathmandu, Lalitpur, Bhaktapur) selected to represent a mix of rural and urban areas where FCHV programs exist alongside alternative CHW models such as community nurses piloted by the Ministry or NGO-sector public-private partnership CHWs (Nepal et al. 2020, DoHS 2024), to serve the objectives of the larger research project that this paper belonged to (see Supplementary Data Appendix S2). Data collection and reporting followed Consolidated criteria for Reporting Qualitative research guidelines (Tong et al. 2007); see complete checklist in Supplementary Data Appendix Table S4.

##### Participants

A convenience sample of purposefully selected stakeholders with expertise on FCHVs and/or federalization were selected, followed by snowball sampling. The final sample comprised 40 informants (Table 2): The majority were female (63%). FCHV union representatives included informants from the Health Volunteers Organisation of Nepal and Nepal Health Volunteers Association, and an international trade union federation, Public Services International (PSI). Half of FCHVs in our sample were aware of or affiliated with unions.

**Table 2.** Informant characteristics.

Category	Number of informants (%)
Informant type	
Ministry of Health and Population officials	4 (10%)
Local government (*‘*palika*’*) policymakers and health administrators	6 (15%)
Public-sector health facility workers (incl. supervisors)	8 (5%)
FCHVs	6 (15%)
FCHV unions	3 (8%)
NGO representatives engaging FCHVs	4 (10%)
International donors and implementation partners	2 (5%)
International and national researchers with longstanding experience evaluating FCHV programs	7 (18%)
TOTAL	**40**
**Gender**	
Male	15 (37%)
Female	25 (63%)
Location: Municipality (district)	
Bhaktapur (Bhaktapur)	6 (15%)
Bhimeshwor (Dolakha)	6 (15%)
Chandragiri (Kathmandu)	6 (15%)
Lalitpur Metropolitan city (Kathmandu)	3 (8%)
Kathmandu Metropolitan city (Kathmandu)	18 (%)
Virtual	1 (3%)

FCHV, female community health volunteer; NGO, non-governmental organization.

##### Definition of a CHW

We considered FCHVs as the only public-sector CHW that meets internationally accepted criteria (WHO 2018b): Although auxiliary nurse midwives (ANMs) and health workers (AHWs) are sometimes classified as CHWs (Rawal et al. 2020, Ghimire and Bhatt 2025), both do largely facility-based work with little outreach, and are often not from the communities they serve (Balakrishnan and Caffrey 2022). Thus, we consider them mid-level workers (Bangdiwala et al. 2010). We however acknowledge that the classification of these workers is contested (Ban et al. 2021, Hodgins et al. 2025).

##### Data collection

Semi-structured interviews (*n* = 26) and focus group discussions (*n* = 5; 2–4 persons/each) were conducted between May and June 2023 using pilot-tested interview guides (see Supplementary Data Appendix S2). Focus groups supplemented interviews to allow for a more natural, ‘empowered’ group setting for sharing information (Burnham et al. 2008). At the end of discussions, participants were offered in-depth interviews to elaborate on their answers. Interviews and focus groups were conducted face-to-face in offices, cafés, or online (one informant), in English or Nepali (58%), and audio-recorded. Participants received written and verbal information on the study’s purpose, voluntary participation, data confidentiality procedures, right to refuse and withdraw, and anonymized use of research findings. Consent was taken in writing or verbally (one participant, recorded on audio device), in Nepali or English. Ethical approval was from the Nepal Health Research Council Institutional Review Committee (May 2023) and the authors’ Institutional Review Committee (May 2023).

##### Data analysis

Interviews were transcribed and translated into English. Thematic content analysis (Braun and Clarke 2022) followed Creswell’s (2018) six steps. The coding scheme was based on inductive (from transcripts) and deductive (from our framework) themes (Fereday and Muir-Cochrane 2006, Elliott 2018). Transcripts were coded using Dedoose software by two researchers, iterating on the codebook as additional themes arose. The final codebook is given in Supplementary Data.

#### Secondary data

##### Document analysis

Interview and focus group data were triangulated with document analysis, to help answer questions arising from primary data, corroborate findings, and reduce potential bias among informants (Bowen 2009, Maxwell 2013). We analysed policies, laws, plans/strategies, briefs, reports, presentations, and articles by federal and local/provincial governments; donors/international development and implementation partners; INGOs; NGOs; global trade union federations (UNI Global Union, PSI); and news media (full list in Supplementary Data Table S5), identified through online searches or recommended by interview participants. Translation of Nepali documents was primarily done by Nepali-speaking coauthors, but some were translated using Google Translate and/or artificial intelligence (Microsoft Copilot) and subsequently verified by Nepali-speaking coauthors.

## Results

We assessed FCHV governance under federalism according to ‘inputs’, ‘processes’ and ‘outputs’ and their sub-dimensions (Table 1). Where possible, we indicate how that dimension changed under federalism. Opportunities and challenges presented by federalism for the six actors are referred to throughout and summarized in Table 3.

**Table 3.** FCHV Governance opportunities and challenges by actor in federal context.

	Opportunities	Challenges
**Local govts (*palikas*)**	Greater autonomy to tailor FCHV programs to local needs Increased fiscal space for FCHVs/community health Some coordination with federal govt on drafting policy (e.g. FCHV retirement).	Some local leaders unfamiliar with FCHVs Federal govt retain much of ownership, constraining ‘decision space’ Variable internal revenue raising capabilities Federal FCHV funding as conditional grants Federal oversight over spending Low intergovernmental transfers from Provinces Capacity gaps in leadership and monitoring Dependent on federal gov. for donor funds Challenges enforcing FCHV selection, retirement, and political activity, and monitoring performance Turnover of younger FCHVs
**Provinces**	Main role: FCHV Training Centres Some role in policy formulation and budgeting (e.g. incentives)	New entity, unclear role in community health Reduced authority and human resources at district satellite offices Leadership capacity shortcomings Provincial Training Centres understaffed and underfinanced; coordination gaps with *palikas*
**Federal Govt (MoHP)**	Minimum educational requirements enable more literate FCHV cadres (successful in urban settings) More robust data reporting	Coordination challenges across disease-specific FCHV programs run by different DoHS divisions, some task duplication Reporting from *palikas* challenged by uneven health facility access to computers & internet Enforcement of federal priorities (e.g. minimum education, retirement, political involvement), challenged by poor enforcement by HMGs Donor funding decreased, expected to decline after 2026 Least Developed Country graduation USAID withdrawal threatens future of planned national FCHV program evaluation
**International donors**	Continued role in funding, planning, drafting policy Represented on federal FCHV Committee Deputed staff at Provinces, discussing direct contracting with *palikas*, Provinces	Reduced representation on federal FCHV Committee post-federalism (7 seats to 1 seat) Shift in responsibility from Ministry’s Family Welfare to Nursing Division posed some challenges Decline in government FCHV funding requests
**FCHVs and unions**	Increased participation in local decision-making, included on *palika* FCHV committee, and local review meetings Strong community recognition; Some elected into local governments Unions have some influence on federal and local policy Federal incentives paid through direct electronic bank transfers Widened role over time, addressing NCDs and social determinants	Unions not consistently included in decision-making; no seat on FCHV committee or local review meetings Unions report accountability fragmentation across three government tiers Low literacy and high age threatens community legitimacy; gov’t literacy programs ineffective Pilots to replace in urban areas Reduced pre-service training (18 to 10 days), minimum education policy challenges enrolling marginalized groups as FCHVs Feelings of inequity from local incentive variation Inadequate training, supervision, role clarity Overburdening
**NGOs**	Represented on federal, provincial & *palika* FCHV committees Involvement in drafting Community Health guideline, provide technical support to MoHP	

DoHS, Department of Health Services; FCHV, Female community health volunteer; HMG, Healthy Mother’s Group; MoHP, Ministry of Health and Population; NCD, non-communicable disease; NGO, Non-governmental organization; USAID, United States Agency for International Development.

Sources: Primary and secondary data.

### Input

Here, we summarize findings for ‘Strategic Vision’ and ‘Participation & Consensus-orientation’.

#### Strategic vision

For this dimension, our assessment indicated that envisioned government roles on paper did not always match with reality, but local planning was emerging.

Prior to 2015, the MoHP was responsible for FCHV policy and budgeting, and District Health Offices for implementation and monitoring (FHD 2010). Although districts reported to Regional Health Directorates, these came under the direct financial management of the MoHP (DoHS 2017). Local governments (Village District Committees, municipalities) had little-to-no role in planning, policy formulation or implementation (Bhandari et al. 2020). Under federalism, *palikas* are tasked with formulating and implementing FCHV policies, adding to incentives and paying half of FCHV health insurance premiums, providing training, monitoring and supervision, and holding twice-yearly local review meetings (NSSD 2019). The federal government sets the overall strategy and the training curriculum, provides conditional grants to *palikas*, and funds dress, transportation, and retirement incentives (DoHS 2025).

**Figure 1.**
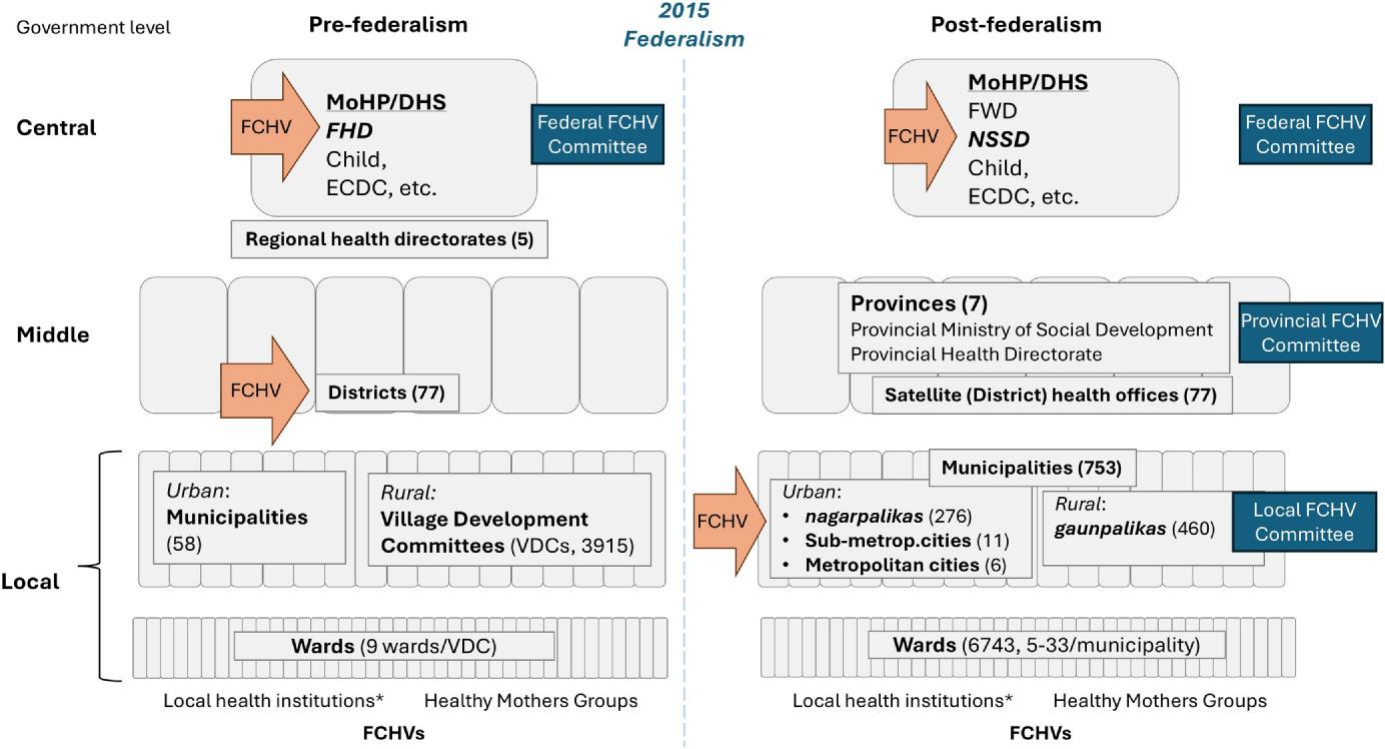
Administrative governance structure of Nepal’s female community health volunteers before and after federalism (2015). Footnotes: *Local health facilities (basic health service centres): Health posts, Community health units, Urban health clinics/centres and Primary health care centres. Arrow denotes division responsible for FCHVs at the national Department of Health (Ministry of Health and Population) level, and at the local level. Abbreviations: DoHS, Department of Health Services; EDCD, Epidemiology and Disease Control Division; FHD, Family Health Department (renamed Family Welfare Division, FWD post-federalism); MoHP, Ministry of Health and Population; NSSD, Nursing and Social Security Division; VDC, Village development committee.

Federalism shifted FCHV responsibility within the Ministry’s Department of Health Services (DoHS) from the Family Health (renamed ‘Family Welfare’) to the new Nursing and Social Security Division (since 2018; Fig. 1, Table 4). The Ministry’s vision for FCHVs was specified in the National FCHV Strategy. Compared with the pre-federal strategy from 2010, the Nursing Division’s 2019 strategy introduced minimum education requirements of grade 10 schooling or basic education in remote areas (FHD 2010, NSSD 2019). Although the supply of FCHVs had remained stable at around 51 000–52 000 nationally for more than a decade, the government predicted 57 000 FCHVs will be needed by 2030 (MEC 2023, Samiti 2023).

**Table 4.** FCHV governance roles for the three government tiers before and after federalism.

Gov’t tier		Before federalism	After federalism/changes since 2015 (if any)
**Central**	**Ministry of Health and Population**	Determines government incentives, FCHV training curriculum.	Determines federal incentives, training curriculum. Policy formulation, monitoring, coordination.
	**DoHS Family Health Div.** *(*renamed ‘Family Welfare Div. 2017/18)	Manage and oversee FCHV program, policy formulation.	Responsibility for some programs in which FCHVs work (e.g. vaccination, family planning, safe motherhood, etc.)
	**DoHS Nursing & Social Security Div.** *(2018-)*	*-*	Main regulatory, policy, budgeting, quality, and coordination body for FCHVs.
	**Other DoHS div.s** (e.g. Epidemiology and disease control, Child health)	Responsible for some programs (e.g. malaria, lymphatic filariasis, HIV, etc.)	(unchanged)
	**Central FCHV Committee**	Policy formulation, review and approval of new programs, implementation support, coordination between programs. Meetings 3/year. Members: various Ministries and agencies/divisions (13 members), donors/INGOs (7). Chair and Secretary from Family Health Division	*Changes:* Meetings semi-annually. Members: Ministry and agency/division representatives (18 members), donors (1), NGOs (1). Chair and Secretary from Nursing and Social Security Division; Family Welfare Division represented (1).
**Mid**	**Regional health directorate**	Coordination, monitoring, guidance/technical assistance to districts	-
	**Provincial Health Training Centre**	-	Basic and refresher training to FCHVs
	**Provincial FCHV Committee**		Policy formulation, review and implement programs, stakeholder coordination, develop training curricula. Semi-annual meetings. Chair and Secretary from Provincial Health Directorate. Other Members: Provincial agencies (8), NGOs (2).
	**Provincial Health Directorate** (under Provincial Ministry of Social Development)		Policy formulation, review programs, implementation, coordination, develop (training) curricula. Monitor FCHV incentives
	**Districts**	**District (public) health offices:** ^a^ Main FCHV responsibility. Implementation, monitoring, report to regional directorate and Family Health Division. Participate in FCHV Review meetings. Establish and monitor district-level FCHV Fund.	**(District-level) health offices:** Manage logistics, supplies, technical assistance to *palikas*. Collate data from *palikas* on FCHVs, report to Provinces and Nursing and Social Security Division.
**Local**	**Local FCHV Committee** (since 2019)	-	Review programs & performance. Stakeholder coordination. Formulate procedures and plans, monitor implementation, regulation. Monitor FCHV Fund. Members: *palika* representatives (3 members), Ward Chairperson (1), FCHV (1), representatives of underserved/marginalized community (2), health-related organisations (1).
	**Local governments**	**Village Development Committee (rural), municipalities (urban):** Operate FCHV Fund. Provide uniforms ‘from time to time’. Provide recognition to well-performing FCHVs in public venues. Join FCHV Review Meeting if invited.	**Municipalities/*palikas*:** Primary FCHV responsibility. Implementation, monitoring, and reporting. Draft guidelines. Provide local incentives in coordination with (district) health office; report to Provincial health directorate and Nursing and Social Security Div. Provide FCHV training. Ensure FCHV supply: 1/1000 people in Terai, 1/600 in hills, 1/150 in mountain regions; financially responsible for additional FCHVs beyond this. Monitor FCHV Fund.
	**Wards** (lowest administrative structure)	Members may be invited to FCHV Review meetings.	Determines working area and number of FCHVs based on health facility recommendations. Members may attend FCHV Review meeting; Chairperson is member of local FCHV committee.
	**Local health facilities** ^b^	FCHV activity monitoring (annual reports). Supervision by AHWs. Supply FCHVs with medicines, materials. Mobilizing FCHVs. Conduct two-day FCHV Review Meetings twice-yearly, attendees: local health workers, local representatives. Collaborate with districts and local government to provide additional support. Support HMGs.	*Changes:* Monthly supervision. Collect FCHV monthly and annual reports. Keep records of FCHV selection and retirement (reported by HMG). Implementation & stakeholder coordination. Provide monthly refresher training.
	**Healthy Mother's Group (HMG)**	FCHV selection, replacement (if takes paid job, inactive, retires, death) and retirement approvals. Evaluates FCHVs’ work, collects FCHV annual reports, submitting to local health facility. FCHV calls monthly meeting and sets agenda. FCHV acts as Secretary of group.	*Changes*: Evaluate FCHVs’ work at least annually, conduct review meeting. Collects FCHV monthly and annual reports. Report on FCHV selection, activities, resignations and replacements, to local health institution. Monitor FCHV Fund. In the event of FCHV vacancy, find replacement within 30 days. FCHV assists Chairperson in holding monthly meetings.

**Sources**: (FHD 2008, 2010, NSSD 2019, DHS Annual Reports 2009–2023, Ban et al. 2021).

AHW, auxiliary health worker (incl. Auxiliary health workers and auxiliary nurse midwives); Div., division; FCHV, female community health volunteer; DoHS, Department of Health Services; HIV, human immunodeficiency virus; HMG, healthy mothers’ group; INGO, International non-governmental organization; MOHP, Ministry of Health and Population; NGO, non-governmental organization.

^a^Prior to 2015 (federalism), 26 districts had District Public Health Offices and 49 had District Health Offices; despite differing nomenclatures, they performed similar functions.

^b^‘Basic Health Service Centres’: Primary Health Care Centers, Health Posts, Community Heath Units and Urban Health Centers. ‘-’ denotes this governance structure did not exist at this time.

Regarding local planning, *palika* leaders admitted federalization had made their work easier and several were formulating FCHV plans and guidelines, which required the Ministry’s approval (SSI_Palika_1, SSI_Palika_4). Although published policies did not yet include FCHV-specific goals (Chandragiri Municipality 2019, Bhaktapur Municipality 2023), several *palikas* had allocated top-up incentives to FCHVs (‘Institutional capacity’).

Unresolved questions of ownership persisted, however: both *palika* and Ministry representatives described program ownership and guardianship falling under the Ministry, with *palikas* mandated to follow their guidelines (SII_Palika_6;SSI_MoHP_3; SSI_MoHP_4). A researcher described that the Ministry developed detailed annual work plans ‘that dictate to the local government what they’re supposed to do’ (SSI_Researcher_6). In 2017 and 2024, the Ministry released guidelines detailing protocols for *palikas*, Provinces and districts for handling FCHV retirement, training, incentive payments, and program delivery (MoHP 2017, 2024).

Provinces were a newly-created administrative structure. Many informants described them as having a more limited and unclear role in community health, with Provincial Health Policies generally copy-pasted from federal plans (SSI_Donor_1; SSI_Researcher_2; SSI_Palika_2). For FCHVs, their main role was to provide training at Provincial Training Centres (DoHS 2021, SSI_Researcher_6). Bagmati had however formulated policies around FCHV incentives, paying the remaining half of their health insurance premium (a role envisioned for *palikas*) and an annual bonus (NSSD 2019; Poudel 2023a, UNI Global Union 2023, 2024, Bagmati MoH 2024).

Federalism reduced the role of districts in FCHV governance, at least on paper. According to guidelines, District health offices operate as satellite outposts for Provinces with their roles limited to coordinating data collection and distributing federal grants from Provinces to *palikas* (MoHP 2017, 2022, 2024, DoHS 2021, SSI_Researcher_6). However, the prioritization of disease-specific programs continued at district, rather than *palika,* levels (MoHP 2017, DoHS 2021, WHO 2023, DoHS 2024, UNICEF 2024). The MoHP also gave districts the joint responsibility (alongside the Ministry’s Nursing division and *palikas*) for addressing challenges such as low utilization of the FCHV Funds and enforcing retirement (DoHS 2023). Thus, districts continue to play some role in FCHV governance in practice.

#### Participation & consensus-orientation

An assessment of mechanisms ensuring participation of actors in decision-making indicated that participation of FCHVs has improved under federalism, while NGOs had an outsized influence in discussions on professionalizing community health delivery.

Federalization enhanced FCHV participation in local decision-making by granting them a seat on *palika*-level FCHV committees—although not on provincial or federal committees (Table 4). Also, because of their social recognition, FCHVs were increasingly elected to local government, enabling them to advocate changes (Singh 2024). At the federal level, however, FCHVs were excluded from decision-making, such as discussions around retirement incentives (Chakma 2022). Ministry representatives described listening to FCHVs’ views during FCHV review meetings and field visits (SSI_MoHP_4, SSI_MoHP_1).

FCHV unions, however, had no official seats on federal, provincial or *palika* FCHV committees, but advocated at all three government levels (SSI_Union_3). While one Ministry representative described a good relationship and regular meetings with unions (SSI_MoHP_4), another was not aware of their existence (SSI_MoHP_2). Union leaders described their relationship with central and *palika* governments as variable (SSI_Union_3, SSI_Union_2) but had succeeded in negotiating raised incentives at federal and *palika* levels (SSI_ Municipal_6; SSI_Municipal_2) and enjoyed good recognition even with parliament members (SSI_NGO_1).

Nationally, only 25%–30% of FCHVs were union-affiliated (PSI 2018, UNI 2024), which was a barrier to negotiating with policymakers (SSI_Union_1).

International donors have participated in FCHV decision-making from the beginning, including planning, funding, and drafting FCHV guidelines and strategies (FHD 2010, Nepal Family Health Program II 2012, Middleton et al. 2013). Under federalism, donors continued their participation in the federal FCHV committee, although their representation was drastically reduced (Table 4); however, they were not represented on Provincial or *palika* FCHV committees.

One area where the government had to mediate between differing viewpoints was around the introduction of parallel, professionalized CHW cadres; here, NGOs played an outsized role. Under federalism, NGO representatives were granted seats on FCHV Federal and Provincial committees—a considerable change from before federalism (Table 4). Two NGOs had piloted professionalized, salaried CHWs with tasks similar to FCHVs but with regular supervision, digital reporting tools, and higher educational requirements, in two districts through a public−private partnership with the Ministry and local governments (Dreisbach 2019). This model was heavily criticized by the central government and USAID as CHWs’ salaries exceeded those of ANMs, making the model ‘unsustainable’ (SSI_Researcher_2). The NGO’s rationale was that professionalized CHWs would be more effective than FCHVs who had reached a ‘ceiling’ beyond which health indicators were not improving (Dreisbach 2019, SSI_NGO_3). The Secretary of Health at the time, who was a board member of one of the NGOs, had sanctioned these pilots (SSI_Researcher_2; Dreisbach 2019).

One of the NGOs had helped draft the national 2021 Community Health Guidelines (NSSD 2021a), which envisioned door-to-door provision of basic health services nationally by community nurses, specifying only a ‘supporting’ role for FCHVs. An NGO representative described this as ‘a milestone’ in their advocacy (SSI_NGO_4, SSI_NGO_3). Based on the NGO’s recommendations, the Nursing Division was piloting community nurse-led care in four municipalities as of 2025 (SSI_NGO_3; Possible Health 2022; DoHS 2025), with the NGO servings as a technical partner (SSI_NGO_3). A Ministry representative described the NGO as the ‘backbone’ of their community health program and admitted they aimed to replace urban FCHVs with community nurses (SSI_MoHP_4; FWD 2021). Although this had not been discussed openly (SSI_NGO_3), in Kathmandu, a FCHV union leader was aware of these intentions (SSI_Union_2).

Regarding participation of civil society actors, federalism introduced a requirement for newly-created *palika*-level FCHV committees to have two members from marginalized groups (NSSD 2019), a considerable change to before (FHD 2010). However, no further findings emerged for civil society actor participation.

### Processes

Here, we review findings for ‘Institutional Capacity’, ‘Accountability & Transparency’, ‘Reporting & Information Systems’, and ‘Rule of Law & Enforcement’.

#### Institutional capacity

Examining the capacities to execute strategic goals, we found that the overall budgetary space of *palikas* had increased, but their budgetary capacity was restrained by continued federal control. Leadership capacity and human resource gaps prevailed sub-nationally, and coordination structures were ineffective.

In line with expectations, the majority of the FCHV budget was distributed to subnational governments during the first 5 years of federalism, and the fiscal space across *palikas* nearly doubled from 990 NPR million to 1 768 NPR million (in nominal terms) (MoHP 2022). FCHV activities also represented a larger share of *palika* health budgets than of provincial and federal budgets (MoHP 2022). This had enabled *palikas* to allocate monthly FCHV allowances and supplement federal retirement allowances. Budget absorption rates (expenditures relative to budgets) for FCHV activities were also higher (92%–100%) than for the health sector overall in Dolakha (71%; Bhimeshwor Municipality 2024). Intergovernmental transfers from the federal government favoured *palikas*, contributing to financial shortfalls at Provincial training centres (SSI_Researcher_2; SSI_NGO_1; SSI_Researcher_1).

The federal government retained substantial control over *palika* spending. Federal FCHV funding was entirely through conditional grants (MoHP 2022). As internal revenue-raising capacity varied widely across *palikas*, some were entirely reliant on federal allocations. A researcher described that: ‘the most powerful person in the municipal administration is… a staff of the central government embedded at the municipality level… to sign off on all expenditures against what’s in the (annual) work plans’ (SSI_Researcher_6). Intergovernmental transfers from Provinces to *palikas* were also typically minimal.

These dynamics also impacted *palikas* ability to afford FCHV supplies. A local health worker lamented: ‘there is no budget for health posts in local Government…We rely on upper levels just to buy (para)cetamol’ (SII_HW_1). USAID’s Suaahara project had supplied Vitamin A capsules and deworming tablets during national campaigns, (HKI/USAID 2022) but the availability of these was severely disrupted following USAID’s funding withdrawal in 2025 (Poudel 2025a).

Leadership capacity gaps were described at *palika* and Provincial levels (SSI_Researcher_1; SSI_Researcher_5). As some Mayoral offices were unfamiliar with the FCHV program and how it operated (Saha and Bhatt 2019), a union leader and NGO representative were actively contacting and educating them on the value of mobilizing FCHVs (SII_NGO_1, SII_Union_1). Donors provided capacity-building to *palika* and Provincial leaders (UNDP 2019, UNESCO 2021, WHO Nepal 2023).

The lack of sufficient human resources was noted at all government levels, but was particularly acute in Provinces (WHO Nepal 2023, SSI_NGO_1, SSI_Researcher_2; SSI_NGO_1; SSI_Researcher_1). A researcher expressed this being a considerable reduction compared to pre-federalisation, (SSI_Researcher_6).

Several informants described coordination gaps across government tiers. In Bagmati, the FCHV training manual budget was distributed to *palikas*, despite Provinces having training responsibility (SSI_Researcher_2). Researchers described instances of representatives of the three government tiers never having met each other (SSI_Researcher_5, SSI_Researcher_1). A Ministry representative had however engaged *palika* leaders in discussions around the retirement policy (SSI_MoHP_4).

Federalism also challenged coordination with donors. A donor representative described that the shift in Ministry responsibility from the Family Welfare to the Nursing Division had led to less government collaboration (SII_Donor_2). Donor flows had reduced in the previous decade. Although some anticipated further reductions with Nepal’s graduation from Least Developed Country status in 2026 (Government of Nepal 2024, SSI_Researcher_1), USAID’s funding cuts in 2025 threatened this transition (Ghimire 2025). *Palikas* were wholly dependent on the MoHP for donor allocations (MoHP 2022), but some donors were negotiating with the federal government to allow direct funding to Provinces and *palikas* (SSI_Donor_1). Others had already deputed staff at Provinces (SSI_Researcher_4; WHO Nepal 2023).

Coordination across disease-specific programs was the task of the federal FCHV committee, a structure replicated at all three government levels under federalism (Table 4; NSSD 2019, FHD 2010, MoHP 2019). Grey literature reports however indicated major coordination challenges across programs (Dreisbach 2019, Balakrishnan and Caffrey 2022). Individual divisions within the DoHS (Table 4) were also described to work a ‘siloed’ fashion (SSI_Donor_2).

#### Accountability and transparency

We show that health system accountability gaps to FCHVs persisted, and while federalism contributed to accountability fragmentation, *palikas* were viewed as more accountable and transparent. FCHVs’ accountability mechanisms to communities were largely ineffective.

Health systems fell short in ensuring FCHVs an enabling working environment. Supervision gaps by facility-based workers—both in quality and quantity—were frequent (SSI_Researcher_2; SSI_Palika_1; SSI_Palika_2), compounded by shortages, low incentives and training gaps particularly in rural areas (Saha and Bhatt 2019, Exemplars in Global Health 2020, Balakrishnan and Caffrey 2022, HKI 2023b). The government had also terminated entry-level ANM and AHW training programs in recent years (Paudel 2019, MEC 2023). FCHV training had been largely donor-funded pre-federalism (USAID 2012, Zullinger 2017). Several informants and a donor report described FCHVs who had not received refresher training in several years (SSI_HW_1, SSI_Union_1; Behavioral Science Center 2023). Where FCHV trainings were reported to be ‘recent’ or ‘frequent’ (SII_HW_1; FDG_FCHV_1_3), these were typically connected to donor programs. For example, USAID had trained approximately half of FCHVs nationwide in nutrition, hygiene and water treatment counselling (HKI/USAID 2022). A donor report also described training as being of low quality (Balakrishnan and Caffrey 2022). While it was unclear whether FCHV training had been exacerbated by the federal transition, one INGO report suggested this was the case (Saha and Bhatt 2019) and a donor described a reduction in government requests towards FCHV training post-federalism (SSI_Donor_2). FCHVs expressed that without refresher training, they forgot training content and felt insecure about service provision (SSI_Donor_2; SSI_FCHV_1).

A FCHV union leader described that federalism had resulted in accountability fragmentation:

We take our concern at the local level and they tell us to go to the provincial level. We do same with provincial level, they… ask us to take our problems to the central government. In the end, we go to the central level, but they tell us to solve at the local levels. It’s like a web of steps … it doesn’t solve our problems but creates more. (SSI_Union_3)

Compared to the federal government, which was subject to frequent reorganization and corruption scandals, *palikas* and Mayors were described as overall more stable, accessible and transparent (SSI_Union_1; SSI_NGO_1).

Several informants reported that incentives did not consistently reach FCHVs, nor did promises of increased incentives always materialise, both at federal and *palika* levels, before and after federalism (SSI_Union_1, SSI_Union_2, SSI_Union_3, SSI_FCHV_1, SSI_HW_1). In 2022, a FCHV union leader described engaging in an eight-month-long battle, backed by the Ministry of Health and Finance and media outlets, to claim full release of their doubled transport allowances, when local health workers had illegally held on to half of the funds (SSI_Union_1). The federal government was addressing opportunities for local skimming by mandating federal incentives should be transferred electronically directly to FCHVs’ bank accounts (MoHP 2024, SSI_Union_1, SSI_FCHV_1).

FCHV’s accountability to health systems and community members were specified in the Code of Ethics they sign upon joining, as being to HMGs and ‘the (federal) ministry and its departments’. This wording was left unchanged post-federalism, rather than delineating accountability to *palikas* (FHD 2010, NSSD 2019). Under federalism, HMGs gained a stronger role in FCHV monitoring, now collecting monthly reporting forms in addition to annual reports, alongside local health facilities and workers (Table 4).

FCHVs also had the opportunity to voice community concerns through their membership in Health Facility Operations and Management Committees. While FCHVs were largely inactive in these committees (Nepal Health Sector Support Programme 2023, Dhungana et al 2024), some had voiced concerns over illegitimate out-of-pocket transportation fees charged to patients during public hearings held by USAID’s Suaahara program (HKI 2023a).

#### Reporting and information systems

Here, we show that the federal government was collecting more data about and from FCHVs, aspiring to digital reporting and evidence-based policy. The upcoming national FCHV evaluation was halted by USAID’s withdrawal in 2025, while local monitoring and evaluation capacities were generally poor.

The Ministry expected health facilities to report data on FCHV demographics, training dates, education level, incentive receipt dates, activity levels, and retirement date into the national FCHV Registry through a web-based system (NSSD 2021b). Perhaps signalling slow reporting, the Ministry had issued requests for this data to Provinces and districts in 2022 and 2024 (Baahrakhari News 2022, MoHP 2024). Complicating matters is that nearly half of health facilities lacked computer and internet access as of 2024 (DoHS 2025).

**Figure 2.**
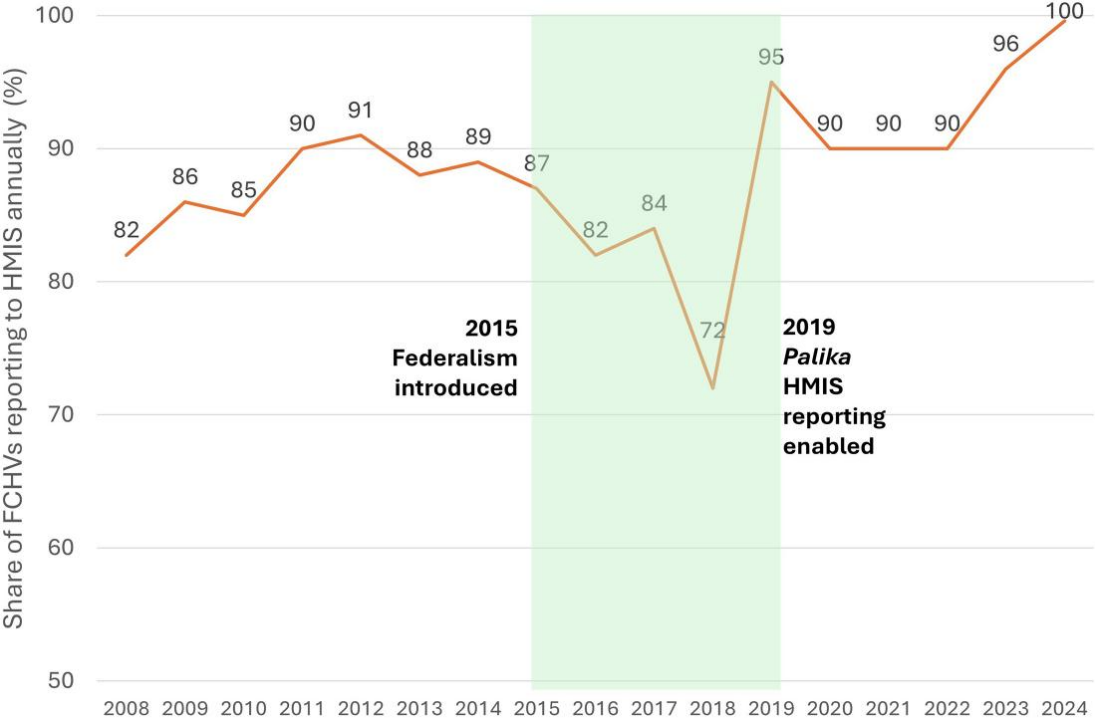
FCHV reporting rates. Share of FCHVs nationally reporting to the government’s Health Management Information System (HMIS) annually. Source: Image created by authors based on data from Department of Health Services Annual Reports 2008–2024.

FCHVs report their activities through a paper-based form. As of 2023, it included 81 items. It had since federalism expanded beyond maternal and child health services, to include seasonal influenza, NCD prevention, mental health counselling, adolescent and elderly patient services, and been converted to written, rather than pictorial format (Jhpiego 2012, Division of Management 2018, NSSD 2021c). Facility workers submit this data online to the federal Health Management Information System, which is also reported to donor agencies. The federal transition period (2015–2019) when reporting duties switched from districts to *palikas*, a dip FCHV in reporting rates (Fig. 2).

The quality of FCHVs’ data reporting was sometimes poor, containing errors (DoHS 2020). This was partly related to low literacy levels, and confusion brought by multiple reporting tools for different programs (Zullinger 2017, DoHS 2021, 2025). Ministry representatives admitted that minimum education requirements were introduced to improve the quality and quantity of data collected by FCHVs (SSI_MoHP_1,SSI_MoHP_2), and to enable their aspirations for digital reporting through mobile devices (NSSD 2019). Although FCHVs already used mobile phones to communicate with community members in some areas, digital literacy was a challenge for older FCHVs (Kathmandu Post 2017, Saha and Bhatt 2019, DoHS 2024). Digital skills training was provided by NGOs in select locations only, contributing to skills gaps (FDG_Researchers_3_1; Saha and Bhatt 2019).

Data-driven policymaking was a policy goal at federal, provincial and *palika* levels (Chandragiri Municipality 2019, MoHP 2023, Bagmati MoH 2024). A researcher however noted that although individual programs were introduced following rigorous pilot-testing, the health sector generally lacked a culture of evidence-based policy (SSI_Researcher_2), exemplified by the 2019 national FCHV guidelines not being adequately informed by findings from the latest national evaluation from 2014 (MoHP 2015).

A new national evaluation on the FCHV program had been planned since 2021 (SSI_Researcher_6; SSI_Researcher_2), the results from which together with results from community nurse pilots (‘Participation’), would inform their decision to strengthen FCHVs or replace them with another cadre (SSI_Palika_4, SII_MoHP_1). Evaluation planning faced delays, however, owing to issues around ownership and design. Although the Nepal Health Research Council wished to take stronger ownership of the evaluation instead of external research organizations, as in past evaluations (SSI_Researcher_6; SSI_Researcher_2; MoHP 2015), some officials claimed that data collected by government agencies was not considered credible by international agencies, stressing the importance of independent evaluations (Poudel 2025b). Although donors had obligated 400 000 USD in funding towards the evaluation in 2023 (SSI_Researcher_6), implementation was halted indefinitely in 2025 when USAID withdrew their funding (Poudel 2025b).

The research, monitoring and evaluation capacity of *palika* staff was noted as poor, with NGOs and donors currently providing the bulk of this (SSI_Researcher_4; WHO Nepal 2023). A researcher emphasized that building such local capacity was necessary (SSI_Researcher_4).

#### Rule of law and enforcement

Here, we demonstrate that HMGs were the main structure for enforcing government guidelines, but were largely ineffective in this task.

HMGs were responsible for ensuring selected FCHVs meet government criteria, both before and after federalisation. Commonly, FCHV replacements were FCHVs’ family members, such as daughters-in-law, or relatives of local elites (SSI_MoHP_4; DoHS 2025, Saha and Bhatt 2019, NSSD 2019), however. Despite minimum education selection criteria in place since 2019, some FCHVs continue to be illiterate, even if university-educated FCHVs were found in cities (DoHS 2025; SSI_MoHP_1, SSI_Palika_3). The Ministry identified such enforcement ‘loopholes’ as a major programmatic challenge (DoHS 2025).

Under federalism, the revised national FCHV strategy introduced a stricter time requirement for HMGs to find a replacement, namely within 30 days (FHD 2010, NSSD 2019). As no sanctions for this policy existed, some locations went without an FCHV as long as one year (NHRC/UNICEF 2023). In addition, enforcing retirement was up to HMGs, with the only sanction for continuing work past age 60 being the cessation of incentives (NSSD 2019; SSI_Palika_1). Suggesting enforcement gaps, many FCHVs continued their role well into their 70 and 80s; In Kathmandu, some even gave false accounts of their age. A union leader noted that deceptive and coercive methods were sometimes used to enforce the retirement policy, particularly illiterate FCHVs (SSI_Union_2).

Chandragiri municipality had successfully retired 22 FCHVs voluntarily in 2022, after increasing the retirement allowance to 100 000 NPR, which made FCHVs feel appreciated for their service (SII_Palika_4).

No enforcement mechanisms existed to ensure FCHVs fulfilled their required duties. Gaps in holding monthly HMG meetings, distributing vitamin A capsules or ensuring childhood vaccinations, were found in government and donor reports (Saha and Bhatt 2019, Dolakha Health Office 2022, NHRC/UNICEF 2023). Although national guidelines require HMGs to remove FCHVs from their post if failing to hold three consecutive HMG meetings (NSSD 2019), it was unclear whether this rule was enforced.

Lastly, it is possible that the fact that national guidelines require FCHVs to act as HMG Secretaries (NSSD 2019) represented a conflict of interest which hindered HMGs in enforcing governmental policies.

### Outputs

Here, we present findings for ‘Responsiveness’, ‘Equity and Equality’, and ‘Efficacy and Efficiency’.

#### Responsiveness

Findings indicated that federal and *palika* governments had expanded FCHVs’ tasks to better respond to local health needs. Government policies were not consistently responsive to FCHV needs, however.

The Ministry had expanded FCHVs’ job duties to address NCDs (NSSD 2019; NHTC 2022; SSI_MoHP_2; SSI_Palika_2). FCHVs in Chandragiri and Sunsari, Jhapa, Dolakha and Kavrepalanchok districts were screening for diabetes, asthma and hypertension (Clean Cooking Alliance 2021, SSI_Palika_2; SSI_HW_1; SSI_NGO_1) and the Ministry had provided special funding to *palikas* to involve FCHVs in mental health screening to respond to rising suicide rates (Poudel 2023b, 2024). Under federalism, FCHVs were also targeting new populations including adolescents, older adults and fathers (Poudel 2019a, NSSD 2021b, NHTC 2022, MoHP 2023, Anmol, 2023).

In addition, FCHVs were involved with social programs such as health insurance enrolment, education and literacy programs, and domestic violence prevention (SSI_MoHP_1; Lohse et al. 2021, Ayer et al. 2024). In the Kathmandu Valley, FCHVs were addressing urban ailments including pollution control, distributing tuberculosis medicines, and identifying missed childhood vaccinations in urban slums (SSI_Palika_2; Poudel 2023c, 2023a).

Other adjustments also enhanced program responsiveness to local needs. In remote settings, the Ministry granted FCHVs wider authority to treat childhood illnesses with antibiotics, and FCHVs faced more relaxed educational requirements (SSI_MoHP_1; SSI_Donor_2). During natural disasters such as hurricanes, the 2015 Earthquakes and the COVID-19 pandemic (DoHS 2020, SSI_FCHV_1; SSI_Palika_5; SSI_FCHV_1), FCHVs have been deployed rapidly by local governments.

These program adjustments were not always responsive to FCHV needs, however. Their tasks now covered 22 service areas, leading to overburdening (Balakrishnan and Caffrey 2022, NHTC 2022, SSI_MoHP_4; SSI_MoHP_1; SSI_NGO_2;SSI_Palika_3;SSI_FCHV_1; FDG_FCHV_1_1). Moreover, FCHVs often refused to retire because of the high status of the position and not feeling appreciated by the retirement incentive (SII_Union_3, SII_Palika_3, SSI_MoHP_1, SSI_Palika_1, SII_FCHV_1). An NGO representative suggested giving retired FCHVs a mentorship role ‘so that they can still feel part of the system’ (SSI_NGO_1). However, retaining younger, more educated FCHVs—as the government envisioned—was challenging. Informants and an INGO report described younger women leaving their post soon after joining for a paid position (FDG_FCHV_1_1, FDG_FCHV_1_2, FDG_FCHV_1_3, SSI_Researcher_2; Exemplars in Global Health 2020).

#### Equity and equality

Here, we show that post-federal policy reforms posed challenges reaching population health equity goals, and FCHVs at times reported unfair treatment.

The MoHP’s guidelines had since before federalism specified selecting women from disadvantaged communities as FCHVs as a priority, and be encouraged to join HMGs (FHD 2010, NSSD 2019). Reaching marginalized populations and health equity were goals also specified in the Provincial health policy (Bagmati MoH 2024).

A researcher described how two post-federal policy changes challenged reaching these goals (SII_Researcher_2). First, the Ministry’s decision to shorten FCHV pre-service training from 18 to 10 in 2020–21 due to increased literacy levels (DoHS 2024) was argued to disadvantage certain ethnic or caste groups such as Chepang or Dalit communities, for whom this may be insufficient. Second, they described that it was difficult to find highly-educated women from such communities to comply with the minimum education requirements. The researcher opined that higher-educated FCHVs, who may more commonly belong to higher caste groups, may not serve women from lower castes. INGO and donor reports gave a mixed picture, finding both gaps in FCHVs reaching marginalized populations (Balakrishnan and Caffrey 2022) and reporting upper-caste FCHVs visited Dalit households, with FCHVs’ ethnic and language composition matching that of their community (Saha and Bhatt 2019).

Regarding equality of treatment among FCHVs, several FCHVs described feelings of unfairness upon learning of increased incentives in other *palikas* (SSI_MoHP_4, SSI_FCHV_1). In one of the Ministry’s community nurse pilot sites, FCHVs had requested incentive raises after learning that community nurses were salaried, despite doing very similar tasks (SSI_Palika_2). Community nurses had, in solidarity requested their managers increase FCHVs’ *per-diem* payments, but to no avail (FDG_HW_1_2, FDG_FCHV_2_2). FCHVs from lower caste groups have reported caste-based (Saha and Bhatt 2019, Balakrishnan and Caffrey 2022, Dhungana 2022), and socioeconomic class-based discrimination in Kathmandu (Poudel 2019b). Our findings did not indicate policy levers to address these challenges.

#### Efficacy and efficiency

Findings suggested that governance challenges hampered meeting strategic goals effectively, with some duplication of activities.

Regular monthly HMG meetings was one of the Ministry’s key Programmatic goals for FCHVs. These were commonly described as ‘inactive’ and mainly focusing on credit savings rather than health topics (SSI_Donor_2, SSI_Researcher_2, SSI_Researcher_6; HKI 2023c, Saha and Bhatt 2019), thus, not achieving their intended purpose (FHD 2008). HMGs were more active in donor-supported areas; for example, USAID provided technical support to HMGs in 42 of 77 districts (Saha and Bhatt 2019, HKI/USAID 2022).

The ineffectiveness of HMGs as a vehicle for improved community health and for enforcing FCHV selection criteria (‘Rule of Law & Enforcement’) was tied to the low legitimacy of these groups and of FCHVs themselves. Young mothers sometimes skipped HMG meetings as they found the health information imparted irrelevant and uninteresting, with their mothers-in-law attending instead (Saha and Bhatt 2019, SII_Palika_4, SSI_MoHP_4; HKI 2023b). Particularly urban residents and higher-educated persons commonly bypassed FCVHs in favour of institutional care, as FCHVs were not seen as knowledgeable (SSI_MoHP_4, SSI_Researcher_2, SSI_Donor_2,SII_FCHV_1; Saha and Bhatt 2019, Save the Children 2017). Urban and Terai (lowlands) region residents were commonly unfamiliar with FCHVs with low service utilization rates (NHRC/UNICEF 2023, DoHS 2025, Saha and Bhatt 2019, Researcher_6). Furthermore, although Ministry guidelines required facility-based workers to assist HMG meetings, they were described to rarely leave their offices (SSI_Researcher_2; SSI_Palika_1; SSI_Palika_2).

The Ministry had attempted to address this legitimacy crisis through minimum educational requirements, retirement incentives and experimenting with nurse-based urban replacements. However, some respondents opined that the federal government had ‘abandoned’ focus on FCHVs, contributing to insufficient institutional support (SSI_Donor_2; SSI_Researcher_4).

Another key lever to achieving the government’s programmatic goals was home visitation. FCHVs however often failed to visit all households containing target populations, both in urban and rural areas (SSI_Palika_4, SSI_FCHV_1; SSI_Researcher_2; NHRC/UNICEF 2023), owing to a combination of FCHVs being unclear on their responsibilities, older-aged FCHVs facing physical mobility challenges, challenging road conditions in remote areas, especially during monsoon season, and lack of transportation (NHRC/UNICEF 2023, SSI_NGO_3; Saha and Bhatt 2019, Behavioral Science Center 2023). A researcher opined that systematic visitation of all households was an ‘ideal on paper’ never realized, apart from locations with substantial donor or NGOs support (SSI_Researcher_6). For example, USAID’s Suaahara program provided household visits to postpartum mothers in coordination with FCHVs (HKI/USAID 2022).

Lastly, some duplication of FCHV activities were reported (Dreisbach 2019, Balakrishnan and Caffrey 2022), related to the largely vertical (disease-specific), donor-funded nature of FCHV programming, and coordination challenges under federalism (‘Institutional capacity’). While federalization presents the theoretical opportunity for *palikas* to remedy fragmentation through horizontal integration at the local level, it was unclear whether this was a policy goal.

## Discussion

Decentralization of CHW governance necessitates defining new processes and roles for actors (Schneider 2019), with federalism representing a form typified by self-rule and shared rule. For the case of Nepal’s FCHVs, we show that the early effects of federalization were characterized by strengthened accountability, transparency, participation and responsiveness of CHW governance, but *palikas* have limited budgetary and administrative decision-space due to continued federal control. Enforcement and efficiency remain among the challenges. While Nepal’s experience with federalization is still evolving, our case study offers lessons to strengthen CHW governance. Using examples from other decentralized contexts, we highlight areas requiring further policy attention.

In Nepal, federalism has enabled *palikas* to tailor FCHV incentives and service areas to better meet FCHVs’ and population health needs, respectively, but not to the degree constitutionally envisioned. The continued reliance on conditional grants—a common phenomenon in recently-decentralized contexts (Rodríguez et al. 2023)—is one aspect constraining local fiscal autonomy. Nepal’s federal government describes them as a ‘temporary modality’ until sub-national governments have built sufficient planning capacity (MoHP 2022). Similarly, the deputing of federal staff at *palikas* to oversee budgetary spending has been described as a ‘knowledge transfer scheme’ ensuring that local leaders acquire the necessary administrative skills (Thaneshwar 2023). Our results confirm previous findings on local leadership and management gaps in Nepal (Thapa et al. 2018, Bhattarai et al. 2022, Wasti et al. 2023, Joshi et al. 2024), thus potentially warranting such transitional measures. Local capacity gaps are however understandable, given that Nepal introduced federalism in one single step, creating subnational governments *de novo*, rather than relying on existing territorial jurisdictions, while concurrently building local institutional capacity. This ‘building a car while driving it’-approach poses greater risks to service delivery than more sequential decentralization reforms introduced over years or decades (Boex et al. 2022). We nonetheless recommend that federal oversight is gradually transitioned in favour of capacity-building of local leaders, a function currently filled by international donors.

Our article adds to the growing body of evidence that CHW representative associations play an emerging role in CHW governance. While FCHV unions were not consistently included in decision-making, they have achieved incentive raises and played a role in holding governments accountable. In India and Pakistan, CHW associations have been crucial in representing CHWs’ interests at central, state, and local levels (Bhatia 2014, Closser 2015, Ved et al. 2019). We support recommendations to involve FCHVs and their representative organizations in decision-making to a greater degree (Panday et al. 2024).

Our study confirms previous findings that Nepal’s federalism is complicated by a lack of role clarity across government tiers, also observed for FCHV governance. This is exacerbated by the sharing of functions across government tiers and local entities, such as FCHV training (provinces, *palikas*) and monitoring (health facilities, HMGs). Given that our findings confirmed that Nepal’s HMGs are largely inactive and not well-attended (Manandhar et al. 2022), more research is needed to elucidate how HMGs enforce policies around selection, retirement and performance monitoring, which are all prerequisites to ensuring that FCHVs fulfil their role towards realizing the constitutional goal of universal access to basic healthcare. Exploring alternative enforcement mechanisms may be warranted.

Shared functions can have negative consequences for accountability. The accountability fragmentation described by unions, also termed ‘bureaucratic gridlock’ (PSI 2024), is exacerbated by the fact that FCHV incentives are now received from all government tiers. Bagmati Province has stepped in to pay the remaining half of FCHVs’ health insurance premiums—an expectation the federal government placed on *palikas*. This makes fiscal sense, given that Provinces have relatively greater internal funding reserves compared to *palikas* (MoHP 2022). We however concur with MoHP recommendations encouraging Provinces to provide conditional grants to *palikas* with limited internal revenues, to even out differentials in fiscal space across *palikas* (MoHP 2022). Indeed, Nepal’s decision to devolve CHW responsibility to local governments stands in contrast to Pakistan and India, where states and territories have the main responsibility.

We also highlight tensions developing countries face when reforming CHW services to better meet the epidemiological shift from communicable to non-communicable diseases. Introducing minimum educational requirements, and the shift in responsibility to the DoHS’ Nursing Division, represent moves towards greater CHW ‘professionalization’ (Schleiff et al. 2021). In Pakistan, educational requirements contributed to rural CHW supply gaps (Folz and Ali 2018); in India, they resulted in selecting non-residents unfamiliar with the community (Singh et al. 2024). Thus, a drive towards CHW professionalization is not always in the interest of service delivery. Hodgins et al. (2021) explicate that: ‘in the minds of many policy-makers … CHWs will no longer be needed once more sophisticated health services are available’. In Nepal, the Ministry’s experimentation with community nurses in semi-urban areas is symptomatic of this. Rather than replacing FCHVs altogether, we recommend *palikas* explore alternatives to encourage the retention of younger, higher-educated FCHVs, such as training opportunities towards being a CHW supervisor or auxiliary nurse, as in Pakistan (Salman and Javed 2017), or India (National Health Mission 2014).

Lastly, we acknowledge that our CHW governance framework relies to a large degree on ‘good governance’ principles, measuring governance *quality*. We recognize that these principles are highly normative, representing democratic values (assumed to be virtuous) defined originally by global institutions such as the World Bank to determine country loan eligibility (Pierre and Peters 2020). We encourage further work to refine CHW governance frameworks, such as using Quality of Governance (e.g. Dahlberg et al. 2025) or Governance Capacity (e.g. Christensen et al. 2016) frameworks. Furthermore, our Donabedian input-process-output adaptation of Siddiqui’s governance framework could be argued to be highly reductionist and ill-equipped to capture health system complexity (Geary 2024). Further adaptations to CHW governance frameworks to better fit specific contexts studied, should be explored.

### Limitations

Bagmati Province and the four study districts have higher-than-average socioeconomic development indicators (MoHP 2022, National Statistics Office 2023a, 2023b). Our findings may thus not be representative of the country at large. Municipalities in our sample were *palikas* classified as ‘urban’ under federalism; however, one was classified as ‘rural’ pre-federalization (when it was a Village Development Committee). While this may have balanced the representativeness of our study, the small sample size precludes generalizations even at the Province level. Although informants were sourced through convenience sampling, thus impacting the generalizability of findings, this was however deemed effective for enabling researcher-participant trust based on ‘most productive relationships’ (Maxwell 2013, p. 134) for a small-scale PhD thesis study with limited resources.

Given that our main interest was the constitutionally-defined role of *palikas* in ensuring basic (primary) health care, Provincial policymakers were not included in our interview sample. However, some findings arose around Provincial roles through informants with prior experience and triangulation through document analysis. Further study sample limitations include the omission of AHWs/ANMs who supervise and train FCHVs; ward representatives, and HMG members. Future studies should include these informant groups, as well as observe FCHV Review meetings, HMG meetings, FCHV committee meetings at federal, provincial and *palika* levels, and Health Facility Operation and Management Committee meetings. We also acknowledge the limitations inherent to translating documents from Nepali to English, as this may introduce an element of misunderstanding across cultural meanings; translations were verified by Nepali co-authors to mitigate this risk.

Lastly, federalization is a *process* that develops over time, and no pre-federal evaluations of FCHV governance existed. Thus, our cross-sectional assessment of FCHV governance at year five of federalism is limited in its ability to draw out pre-post differences in FCHV governance. We hope that our analysis can serve as an ‘early measure’ of the impact of federalization on FCHV governance, sparking future studies to investigate changes over time.

## Conclusion

We assessed CHW governance in Nepal, showing that federalization thus far is characterized by strengthened participation, responsiveness, accountability, and transparency to some extent, while gaps remain around institutional capacity, enforcement and efficiency. Continued federal control of local fiscal and administrative procedures reflects that local ‘decision space’ is not yet realized as envisioned by the 2015 Constitution. Further capacitating local leaders while loosening federal control is required to enable meeting constitutional goals of universal health access and good governance.

## Supplementary Material

czaf088_Supplementary_Data

## Data Availability

The primary data collected for this article (interviews, focus groups), cannot be shared publicly to protect the privacy of individuals who participated in the study. All documents reviewed were in the public domain (online) at the time of writing, and are referenced.

## Reflexivity statement

The authorship team represents diversity across gender identity, seniority, citizenship and professional background. This article includes five authors who identify as women and one who identifies as a man. Four of the authors are early career researchers, and two are in senior positions. At the time of conducting fieldwork, three authors were affiliated with Nepali institutions, and three with European or American institutions. At the time of drafting the article, all authors were affiliated with high-income country institutions in the Global North due to transition of Nepal-based team members to pursue higher education goals. Authors represent a mix of academic, governmental and non-governmental institutions, across both public and private sectors. Four of the authors are persons are citizens and/or have been long-term residents of Nepal, and three authors are citizens of high-income countries. One team member has experience working for Nepal’s Ministry of Health, two have experience working for the Nepal government’s autonomous Research Council, while two members have experience working for a private health organization implementing a professional NGO-funded CHW model in rural Nepal. Collectively, the research team have expertise in public health, epidemiology, clinical medicine, anthropology, informatics, health policy analysis, health management and social science. All authors have experience with qualitative research and fieldwork.
