# Supplementary material for: Assessment of community health workforce governance in federal Nepal
Source: Health Policy Plan. 2026 Jun 29;41(Suppl 1):i17–37. doi: 10.1093/heapol/czaf088 (PMC13311675; doi:10.1093/heapol/czaf088)
Supplement: czaf088_Supplementary_Data [file czaf088_supplementary_data.zip › Supplementary file_REV_Sep25_CLEAN.docx]

Supplementary Data (Appendices)

***Appendix 1. Creation of CHW governance framework from existing literature***

We categorized governance features into: **inputs**, **processes**, and **outputs**, as per the health systems framework of Baez-Camargo & Jacobs (2011). Below we show the overlap between governance sub-dimensions across Siddiqui’s (2009) and more recent frameworks on health workforce (human resources for health) or community health system governance used to update the Siddiqui framework (Chen et al. 2021; Dieleman, et al. 2011; Kaplan et al. 2013; Lim and Lin 2021; Martineau et al. 2022; Sonderegger et al. 2021). The table also shows which dimensions were excluded and reasons for their exclusion.

**Table A1. Comparison of governance dimensions from source health system, community health system and health workforce governance frameworks**

| **Siddiqui 2009** | **Lim & Lin 2021** | **Dieleman 2011** | **Kaplan 2013** | **Sonderegger 2021** | **Martineau 2022** | **Chen 2021** |
| --- | --- | --- | --- | --- | --- | --- |
| **Health system governance**  Health system governance principles   - Strategic vision - Participation and consensus-orientation - Rule of law - Transparency - Responsiveness - Equity and inclusiveness - Effectiveness and efficiency - Accountability - Intelligence and information - Ethics | **Health workforce governance**  *Dimensions of governance **  *Fundamental values:*   - Control of corruption - Democracy - Human rights - Ethics and integrity - Conflict preservation - Public good - Rule of law   *Sub-functions:*   - Accountability - Partnerships - Formulating policy/strategic direction - Generating information/ intelligence - Organizational adequacy/ system design - Participation and concensus - Regulation - Transparency   *Outcomes:*   - Effectiveness - Efficiency - Equity - Quality - Responsiveness - Sustainability - Financial and social risk protection - Improved health | **Human resources for health governance**  *Four dimensions of governance*  *Performance:*   - Efficiency and effectiveness, capacity to implement - Ethics and respect (incl. For citizens) - Intelligence, information, evidence, M&E - Policy objectives vs. Organizational structure capacity to implement, decentralization - Strategic vision, leadership, direction, decision-making process   *Equity and equality:*   - Fairness, equity, inclusiveness, responsiveness   *Partnerships and participation:*   - Consensus-orientation, coalition, partnership - Legitimacy, voice, participation   *Oversight:*   - Accountability - Regulation - Rule of law, enforcement (incl. corruption control) | **Human resource governance**  *8 governance principles*   - Information - Accountability - Strategic vision - Transparency - Efficiency - Equity - Responsiveness - Voice and participation | **Health workforce planning & policymaking logic model**  *Contextual factors*:   - Economic - Social, legal, environmental, epidemiological - Political   *Health system factors*:   - HRH system governance ** - HRH policy formulation and implementation   *Health workforce processes*:   - Production (pre-service training) - Entry - Maintenance and performance - Enabling environment - Exit   *Health workforce outcomes*:   - Availability - Will do - Can do   *Health system outcomes*:   - Coverage - Clinical quality - Efficiency - Responsivenvess | **Health workforce governance**  *Dimensions of HRH governance*   - Accountability - Leadership - Partnership - Ownership - Formulating policy/ Strategic direction - Generating information / intelligence - Organizational adequacy/ system design - Participation and consensus - Sustainability - HRH performance - HRH literacy | **Community Health Systems Reform Cycle**  *Reform Cycle Stages*   - Problem prioritization - Coalition building - Solution gathering - Design - Readiness - Launch - Governance *** - Management and Learning |

**Footnotes:** * Adapted from Barbazza E, Tello JE. A review of health governance: definitions, dimensions and tools to govern. Health Policy. 2014;116(1):1–11. ** Governance Definition: ‘*Leadership, processes, and capacities for governing HRH systems, incl. individual, organizational, and systemic capacity for management and decision-making; collaboration and coordination within and across sectors and ministries for multisectoral action; transparency and accountability to government and communities; and corruption within the HRH and broader health systems*.’ *** Governance definition: ‘*Stakeholders establish a project governance framework, which includes key leadership and decisionmaking bodies, clear roles and responsibilities, and explicit decision rights. It is also critical to establish processes for risk and issue management; stakeholder engagement; and cross-functional communication. As the program evolves, actors monitor and assess progress to advance clear decision making and address critical issues or challenges’*.**Table A2. Final CHW governance framework constructed from existing frameworks on health system, community health system and health workforce governance, showing dimension overlap**

|  |  |  | **Siddiqi 2009** | **Lim & Lin 2021** | **Dieleman 2011** | **Kaplan 2013** | **Sonderegger 2021** | **Martineau 2022** | **Chen 2021** |
| --- | --- | --- | --- | --- | --- | --- | --- | --- | --- |
|  |  | **Framework country/geographical focus** | LMICs; validated for Pakistan | Global | LMICs | Applied to 20 countries (Caribbean, Africa, South America, Europe and Southeast Asia) | LMICs | Applied to Malawi, Nepal, Sudan | LMICs |
|  |  | **Framework based on primary empirical data** | - | - | Yes (16 case studies) | - | - | - | Yes (Bangladesh, Democr. Repub. of Congo, Haiti, Kenya, Liberia, Mali, Uganda) |
|  |  | **Framework based on systematic/literature review** | - | Yes | Yes | Yes | Yes | - | Yes (desk review) |
|  |  | **Framework adapted from published frameworks** | Yes (UNDP good governance, WHO, World Bank, PAHO) | Yes | Yes | Yes | Yes | Yes | Yes |
|  |  | **Framework governance focus** | Health system | Health workforce governance | Health workforce governance | Health workforce governance | Health workforce planning and policymaking | Health workforce governance | Community Health Systems Reform |
| Dimensions | **Categories** | *Sub-categories* |  |  |  |  |  |  |  |
| Included | **Inputs** | **Strategic vision** | v | v | v | v |  | v | v |
|  |  | **Participation** | v | v | v | v | v | v | v |
|  |  | **Consensus orientation** | v | v | v | v | v | v | v |
|  | **Processes** | **Institutional capacity** | v |  | v | v | v | v | v |
|  |  | **Accountability** | v | v | v | v | v | v | v |
|  |  | **Transparency** | v | v | v | v | v |  | v |
|  |  | **Rule of Law & Enforcement** | v | v | v |  |  |  |  |
|  |  | **Reporting & Information systems** | v | v | v | v | v | v | v |
|  | **Outputs** | **Responsiveness** | v | v | v | v | v |  | v |
|  |  | **Equity & Equality** | v | v | v | v | v |  | v |
|  |  | **Efficiency, Efficacy, Performance** | v | v | v | v |  | v | v |
| Excluded (reason excluded) | | **Ethics** (difficult to measure/define; aspects incorporated under Equity) | x | x | x |  |  |  |  |
|  |  | **Human rights** (difficult to measure/define) |  | x |  |  |  |  |  |
|  |  | **Regulation** (incorporated into Institutional design) |  | x | x |  |  |  |  |
|  |  | **Control of corruption** (incorporated into Rule of Law & Enforcement as per Dieleman et al., 2011) | x | x | x |  | x |  |  |
|  |  | **Quality** (incorporated into Efficiency, Efficacy & Performance) |  | x |  | x |  |  |  |
|  |  | **Sustainability** (touched upon in Efficiency, Efficacy & Performance) |  | x |  |  |  | x |  |
|  |  | **Improved population health** (incorporated into Efficiency, Efficacy & Performance) |  | x |  | x |  |  |  |
|  |  | **Democracy** (difficult to measure/define; less relevant to CHWs) |  | x |  |  |  |  |  |
|  |  | **Conflict preservation** (difficult to measure/define; less relevant to CHWs) |  | x |  |  |  |  |  |
|  |  | **Public good** (difficult to measure/define; already relevant for public-sector CHWs in Nepal) |  | x |  |  |  |  |  |
|  |  | **Integration** (difficult to define/measure) |  |  |  |  |  |  | x |

**Sources:** Siddiqui et al., 2009; Chen et al. 2021; Dieleman, et al 2011; Kaplan et al. 2013; Lim and Lin 2021; Martineau et al. 2022; Sonderegger et al. 2021; Baez-Camargo et al., 2011 (see main manuscript for full references).

**Footnotes**: ‘v’ indicates that this dimension was included in the original source framework, and our final analytical framework. ‘x’ indicates dimension was included in the original source framework, but not in our final analytical framework.

**Appendix 2. Interview topics**

Table A2 shows interview topics by participant group. Interview guides were developed separately for each of the seven participant groups (available by request from authors). Interviews followed a semi-structured format, with open-ended questions at the start followed up with increasingly close-ended follow-up questions to clarify responses and ensure that all topics of interest were addressed, where needed. Interview guides were pilot tested prior to implementation in the field, to ensure understandability as well as applicability of the tool to answering the research questions. As the study formed part of a larger research study (‘*Reforming Community-Based Health Care Workforce Policy in Nepal: Current Challenges and Opportunities in the context of Federalization’*), topics shown here were more broadly focused than the topics touched upon in the current paper. In addition, private-sector informants and community health nurses and NGO-based CHWs were excluded from the current study, seeing as their inclusion as participants in the larger project was motivated to answer a different set of research questions than those studied in this paper.

**Table A3. Interview topics by participant group**

|  | Stakeholder interviews: Key Informant Interviews | | | | |
| --- | --- | --- | --- | --- | --- |
|  | **Natl**  **govt** | **Local govt** | **FCHV unions** | **Private sector (NGO, hospitals)** | **Researchers (INGO, donor, academics)** |
| FCHV/CHW relevance for future hcare sys. | **✔** | **✔** |  | **✔** | **✔** |
| FCHV payment policies incl. retirement | **✔** | **✔** | **✔** |  |  |
| FCHV union-gov interactions, views, influence | **✔** | **✔** | **✔** |  | **✔** |
| Federalisation natl-local govt int’act, division | **✔** | **✔** |  |  | **✔** |
| CHW PPP/pilot govt-private interaction & continuation | **✔** | **✔** | **✔** | **✔** |  |
| Alternative cadres: Cadres represented (ANMs, CHWs, CHNs), current goals of programs | **✔** | **✔** |  |  |  |
| Future of CBHC delivery in Nepal | **✔** | **✔** |  | **✔** | **✔** |
| Paid v. unpaid CHW cadre cultural acceptability | **✔** |  | **✔** |  | **✔** |
| Personal recommendations to strengthen CBHC in Nepal | **✔** | **✔** | **✔** | **✔** | **✔** |
|  | In-depth interviews & focus group discussions | | | | |
|  | **FCHVs** | | | **CHWs/CHNs** | |
| Motivation to join |  | **✔** |  | **✔** | |
| Scope of work (SOW) |  | **✔** |  | **✔** | |
| Views on current payment vs. SOW |  | **✔** |  | **✔** | |
| Other income sources |  | **✔** |  | **✔** | |
| FCHV-CHW interaction |  | **✔** |  | **✔** | |
| Union engagement, views |  | **✔** |  | **✔** | |
| Continuation |  | **✔** |  | **✔** | |
| Retirement policy |  | **✔** |  |  | |

Abbreviations: ANM, auxiliary nurse midwife. CBHC, community-based health care system. CHN, community health nurse (MoHP pilots). CHW, community health worker (private sector/NGO). FCHV, female community health volunteer. INGO, international non-governmental organization. NGO, non-governmental organization. PPP, private-public-partnership. SOW, scope of work.

**Appendix 3. Coding scheme on Dedoose for primary data (interviews, focus groups)**

The following codes from Dedoose software were selected for writing the code memos on CHW Governance using dimensions in our framework.

**Table A4. Codes and code counts from Dedoose**

| **Code name** | **Code count (# text excerpts)** |
| --- | --- |
| **National government** | 23 |
| National health plans, policies | 34 |
| FCHV Govt relations | 93 |
| Natl Gov - INGO-Donor Relations | 28 |
| Natl Govt - NGO relations | 21 |
| Natl gov - Local gov relations | 57 |
| **Provincial government** | 47 |
| **Local government (palika, Ward, district):** Incl. FCHVs relations, NGO relations | 173 |
| **FCHVs** |  |
| FCHV Accomplishment | 37 |
| FCHV Accountability & Reporting | 20 |
| FCHV Age & Generation gap | 28 |
| FCHV Areas of Responsibility | 114 |
| FCHV Capacity building & Training | 48 |
| FCHV Challenges | 13 |
| FCHV Eligibility & Selection Policy | 68 |
| FCHV Future | 175 |
| FCHV-Health system relation | 37 |
| FCHV History | 15 |
| FCHVs' Leadership roles | 24 |
| FCHV Management | 16 |
| FCHV Motivation | 30 |
| FCHV National Evaluations | 28 |
| FCHV NGO relations | 23 |
| FCHV Other work & income | 22 |
| FCHV Payment | 212 |
| FCHV Performance & Evaluation | 63 |
| FCHV Retirement policy | 79 |
| FCHV Status in community/society | 31 |
| FCHV work burden | 30 |
| FCHV – Healthy Mothers Group | 23 |
| **Community members** |  |
| Community - Cultural and Social Change | 26 |
| Community - FCHV relations & views | 62 |
| **Health System (government)** | 49 |
| Community health systems & auxiliary health workers | 96 |
| **International donors & INGOs** | 25 |
| Intl donors/INGOs - FCHV relations | 14 |
| **NGOs engaging FCHVs** | 79 |
| **FCHV unions/associations (HEVON, NEVA)** | 62 |
| Natl Govt-FCHV union relation | 21 |
| Local gov-FCHV union relation | 21 |
| Union - FCHV relation | 15 |
| Union Impact on policy | 24 |
| **Trade union federations (GEFONT, NTUC)** | 19 |
| International/global TUFs (PSI, UNI) | 12 |
| **Governance challenges** | 10 |
| **NCDs & epidemiological transition (diabetes, hypertension, mental health, etc)** | 59 |
| **Evidence-based policy** | 87 |
| **Federalism & decentralization** | 45 |
| Federalism impact | 71 |
| Federalism issues & challenges | 22 |
| **Health prioritization in development** | 16 |
| **Rural - urban differences** | 55 |
| **Volunteerism vs. paid workers** | 112 |
| **Professionalization / Institutionalization, Medicalization** | 28 |
| **Catastrophic events** |  |
| COVID-19 | 30 |
| Earthquake 2015 | 6 |
| **Electronic & digital health** | 8 |
| **Unmet needs vs. Policy** | 3 |

**Footnotes**: Parent codes shown in bold; child codes shown in non-bold, indented format. Where no ‘code count’ is shown for parent code (bold), indicates that only the child codes shown below, were analyzed.

**Abbreviations**: FCHV, female community health volunteer; GEFONT - General Federation of Nepalese Trade Unions; HEVON - Health Volunteers Organisation of Nepal; INGO, International non-governmental organization; NCDs, Non-communicable diseases; NEVA - Nepal Health Volunteers Association; NGO, Non-governmental organization; NTUC - Nepal Trade Union Congress; PSI - Public Services International; TUF - Trade union federation; UNI - UNI Global Union.

**Appendix 4. COREQ Checklist**

Below we show the Complete COREQ (Consolidated criteria for Reporting Qualitative research) Checklist, indicating where the item is reported in the main manuscript, and a clarifying description.

**Appendix Table A5. COREQ Checklist**

| **#** | **Item / Topic** | **Reported in manuscript? If yes, where** | **If no, additional description** |
| --- | --- | --- | --- |
|  | **Domain 1: Research team and reflexivity** | | |
|  | *Personal characteristics* | | |
| 1 | Interviewer/facilitator |  | First and second authors and two research assistants |
| 2 | Credentials | Yes: ‘Reflexivity Statement’ | *Clarification on credentials for author team*: medicine (n=1, MBBS); PhD (n=2); Nursing (n=1, BSc); Public Health (n=3, MPH); Master of Healthcare Management (n=1).  *Additional clarification for research assistants not listed as authors but engaged in data collection and/or interview transcription/translation*: Bachelor’s degree in Public Health (n=3). |
| 3 | Occupation | Yes: ‘Reflexivity Statement’ | *Clarification for author team*: PhD student (n=1), research assistant/student (n=1), Professor (n=1), Senior Advisor (n=1), Program Lead (n=1), Health Service Coordinator (n=1). *Note:* Following data collection completion and during results write-up, three authors transitioned to full-time postgraduate studies.  *Research assistants (non-authors)*: student (n=1), Public health officer (n=2). |
| 4 | Gender | Yes: ‘Reflexivity Statement’ | *Clarification for research assistants (non-authors):* female (n=1), male (n=2). |
| 5 | Experience and training | Yes: ‘Reflexivity Statement’. |  |
|  | *Relationship with participants* | | |
| 6 | Relationship established | Yes: Discussion - Limitations | *Clarification*: Most informants, such as most health workers, NGO informants, and Ministry representatives were approached by research team members based at Dhulikhel Hospital Community Health Department, Kathmandu University School of Medicine. Most other informants, including international donors, FCHV union representatives and researchers, were sourced through other research team members and their contact networks. |
| 7 | Participant knowledge of the interviewer | Yes: Discussion - Limitations | *Clarification*: Although sourced through existing contacts, most informants did not have a pre-existing relationship with the interviewer; such a situation arose only for Dolakha district informants. During collection of informed consent, informants were handed a printed Participant Invite Letter outlining the institutions responsible for the study, the study aims and research questions, as well as specific questions or topics the researchers were interested in asking this specific informant group. |
| 8 | Interviewer characteristics | - | The first author (PI, PhD student) was physically present in all interviews (and conducted all English-language interviews); their motivations and interest in the research topic was outlined in the Participant Invite Letters. During Nepali-language interviews, the interviewer introduced their job title and affiliation at the beginning of the interview. |
|  | **Domain 2: Study design** | | |
|  | *Theoretical framework* | | |
| 9 | Methodological orientation and Theory | Yes: Methods – ‘Analytical Framework’, and ‘Data and analysis’ |  |
|  | *Participant selection* | | |
| 10 | Sampling | Yes: Methods – Data and analysis – Participants |  |
| 11 | Method of approach | - | Telephone or email |
| 12 | Sample size | Yes; Table 2 |  |
| 13 | Non-participation | - | 1 person, no reason given |
|  | *Setting* | | |
| 14 | Setting of data collection | Yes: Methods – Data and analysis – Data collection |  |
| 15 | Presence of non-participants | - | No, except for 1 virtual interview with an international researcher which included other researchers with expertise and interest in the topic for another project. |
| 16 | Description of sample | Yes: Table 2, and Methods – Data and Analysis – Data collection. |  |
|  | *Data collection* | | |
| 17 | Interview guide | Yes: Appendix 2 shows interview topics for the; full interview guides are available by request by authors. |  |
| 18 | Repeat interviews | - | Not done |
| 19 | Audio/visual recording | Yes: Methods – Data and analysis – Data collection |  |
| 20 | Field notes | - | Yes |
| 21 | Duration | - | 21-76 minutes |
| 22 | Data saturation | - | The original intent (per protocol) was to collect data until data saturation but because of time and logistical issues, this was not done during actual data collection. |
| 23 | Transcripts returned | - | No |
|  | **Domain 3: Analysis and Findings** | | |
|  | *Data analysis* | | |
| 24 | Number of data coders | Yes: Methods – Data analysis |  |
| 25 | Description of the coding tree | Yes: Appendix 3 |  |
| 26 | Derivation of themes | Yes: Methods – Data analysis |  |
| 27 | Software | Yes: Methods – Data analysis |  |
| 28 | Participant checking | - | No |
|  | *Reporting* | | |
| 29 | Quotations presented | Yes (illustrative participant quotes presented)  Yes (each quotation identified with randomized participant number) |  |
| 30 | Data and findings consistent | Yes: Results |  |
| 31 | Clarity of major themes | Yes: Results (*a priori* specified analytical framework) |  |
| 32 | Clarify of minor themes | - | No. Reason: Word count limitations and because of *a priori* specified analytical framework. Research project gave rise to several research papers, where other themes arising from this analysis are explored in greater detail. |

Footnotes: ‘-‘ refers to item not appearing in main manuscript; these are specified in the ‘additional description’ column.

Table adapted from: Tong et al., 2007

**Appendix 5. List of documents reviewed**

**Appendix Table A6. List of documents reviewed by organization, type and year**

| # | **Organization (Division)** | **Type** | **Document name** | **Year** |
| --- | --- | --- | --- | --- |
|  | **National policy documents** | | | |
| 1 | Ministry of Health, Department of Health Services - FHD | Strategy | 2003 National Revised FCHV strategy (incl. FCHV Code of Ethics) | 2003 |
| 2 | Ministry of Health, Department of Health Services - FWD | Strategy | 2010 FCHV strategy | 2010 |
| 3 | Ministry of Health, Department of Health Services - NSSD | Strategy | 2019 FCHV strategy | 2019 |
| 4 | Ministry of Health, Department of Health Services - NSSD | Code of Ethics | FCHV code of ethics | 2019 |
| 5 | Ministry of Health, Department of Health Services (DHS) | Report | DHS Annual Report 2080-81 (2023-2024) | 2025 |
| 6 | Ministry of Health, DHS | Report | DHS Annual Report 2079-2080 (2022-23) | 2024 |
| 7 | Ministry of Health, DHS | Report | DHS Annual Report 2078-88 (2021-2022) | 2023 |
| 8 | Ministry of Health, DHS | Report | DHS Annual Report 2077-78 (2020-2021) | 2022 |
| 9 | Ministry of Health, DHS | Report | DHS Annual Report 2076-77 (2019-2020) | 2021 |
| 10 | Ministry of Health, DHS | Report | DHS Annual Report 2075-76 (2018-2019) | 2020 |
| 11 | Ministry of Health, DHS | Report | DHS Annual Report 2074-75 (2017-2018) | 2019 |
| 12 | Ministry of Health, DHS | Report | Glimpse of Annual Report DHS 2073/74 (2016/17) | 2018 |
| 13 | Ministry of Health, DHS | Report | DHS Annual Report 2072-73 (2015-2016) | 2017 |
| 14 | Ministry of Health, DHS | Report | DHS Annual Report 2071-72 (2014-2015) | 2016 |
| 15 | Ministry of Health, DHS | Report | DHS Annual Report 2070-71 (2013-2014) | 2015 |
| 16 | Ministry of Health, DHS | Report | DHS Annual Report 2069-70 (2012-2013) | 2014 |
| 17 | Ministry of Health, DHS | Report | DHS Annual Report 2068-69 (2011-2012) | 2013 |
| 18 | Ministry of Health, DHS | Report | DHS Annual Report 2067-68 (2010-2011) | 2012 |
| 19 | Ministry of Health, DHS | Report | DHS Annual Report 2066-67 (2009-10) | 2011 |
| 20 | Ministry of Health | Policy | Terms of Reference (स्वास््म तथा जनसॊख्मा भन्त्रारम) | 2024 |
| 21 | Ministry of Health, DHS - NSSD | Strategy | National Nursing and Midwifery Strategy 2020 - 2030 | 2022 |
| 22 | Ministry of Health, DHS - NSSD | Guideline | Community Health Program Guidelines (Samudayik Swasthya Karyakram Nirdeshika), 2078 | 2021 |
| 23 | Ministry of Health and Population, National Health Training Center (NHTC) | Training Manual | Female Community Health Volunteer Basic Training Manual | 2022 |
| 24 | Ministry of Health, DHS - NSSD | Training Manual | Continuing Capacity Building Materials for Women Health Volunteers 2078 | 2021-22 |
| 25 | Ministry of Health and Population | Strategy | Nepal Sector Strategic Plan 2023-2030 | 2023 |
| 26 | Ministry of Health and Population, Policy Planning and Monitoring Division | Report | Health Sector Budget Analysis: First Five Years of Federalism | 2022 |
| 27 | Ministry of Health and Population – DHS – FHD | Report | Female Community Health Volunteer National Survey Report 2014 | 2015 |
| 28 | Ministry of Health and Population | Strategy | Nepal health sector strategy implementation plan 2016-2021 | 2017 |
| 29 | Ministry of Health and Population | Strategic plan | Nepal Health Sector Programme-2 IMPLEMENTATION PLAN 2010-2015 | 2010 |
| 30 | Ministry of Health and Population – DHS - NSSD | Guideline | Guidelines for the implementation of activities under the conditional grant for health programs to be operated by the province 2081/82 | 2024 |
| 31 | Ministry of Local Development | Report | Policy Paper On Decentralization (NDF-2002) | 2002 |
| 32 | Ministry of Health and Population | Guideline | Guidelines for Implementing Health Programs at the Local Level 2074-75 | 2017-18 |
| 33 | Government of Nepal | Regulation | Public Health Service Regulations, 2077 | 2020 |
| 34 | Government of Nepal | Law (Act) | The Public Health Service Act, 2075 | 2018 |
| 35 | Government of Nepal | Law (Act) | The Local Governance (Local Government Operation) Act, 2074 | 2017 |
| 36 | Government of Nepal | Law (Act) | Federation, Province and Local Level (Coordination and Inter-relation) Act, 2077 | 2020 |
| 37 | Government of Nepal | Constitution | Constitution of Nepal 2072 | 2015 |
| 38 | Ministry of Health and Population - Policy Planning and Monitoring Division, and and DFID/NHSSP | Report | Budget Analysis of Health Sector 2019 | 2019 |
| 39 | Ministry of Health and Population – DHS – Management Division | Report (Fact Sheet) | Nepal health fact sheets 2024 | 2024 |
| 40 | Ministry of Health and Population | Report | Progress of Health and Population Sector 2022/23 (2079/80 BS) NATIONAL JOINT ANNUAL REVIEW REPORT | 2023 |
| 41 | Ministry of Health and Population – DHS – FHD | Guidelines (42) | Female community health volunteer Fund Operational Guidelines (unofficial translation) 2065 | 2008 |
| 42 | Ministry of Health and Population / New ERA / USAID | Report | Nepal Demographic and Health Survey 2022 | 2023 |
| 43 | Government of Nepal / WHO Nepal country office | Strategic plan | Multisectoral Action Plan for the Prevention and Control of Non Communicable Diseases (2014-2020) | 2015 |
| 44 | Ministry of Health and Population | Strategic plan | Multi-sectoral Action Plan for Prevention and Control of NCDs 2021-2025 | 2022 |
| 45 | Ministry of Health and Population – DHS – FWD | Strategy | Nepal Safe Motherhood and Newborn Health Road Map 2030 | 2019 |
| 46 | Ministry of Health and Population – DHS – Division of Management | Report (Directory) | Health Management Information Systems: Directory of Records and Reports, 2075 (second edition) | 2018 |
| 47 | Ministry of Health and Population – DHS – NSSD / Hello World IT Solution Pvt | User Manual | A Comprehensive User Manual On FCHV Registry | 2021 |
| 48 | Ministry of Health and Population – DHS – FWD | Strategy | Strategy for Skilled Health Personnel and Skilled Birth Attendants 2020-2025 | 2021 |
| 49 | Ministry of Health and Population | Strategy | National Human Resources for Health (HRH) Strategy 2021-2030, Nepal | 2021 |
| 50 | Ministry of Health and Population | Policy | National Health Policy 2071 | 2014 |
| 51 | Ministry of Health and Population | Policy | National Health Policy 2076 | 2019 |
| 52 | Ministry of Health and Population - DHS - Child Health Division | Report (Evaluation) | Assessment of the Community-based Newborn Care Package | 2012 |
| 53 | Government of Nepal - Medical Education Council | Report | Health Manpower Projection in Nepal 2079-2087 (2023-2030) | 2023 |
| 54 | Ministry of Health and Population | Guideline | Nepal Health Human Resouce Management Information System Operation Procedures 2081 | 2024/2025 |
| 55 | MOHP/ New ERA / ICF / NHSSP (UKAid) | Report | Nepal Health Facility Survey 2015 | 2017 |
| 56 | New ERA / MOHP / ICF | Report | Nepal Health Facility Survey 2021 Final Report | 2022 |
|  | **Subnational government documents** | | | |
| 1 | Bagmati Province, Ministry of Health | Policy | Bagmati health policy 2081 (2024) | 2024 |
| 2 | Chandragiri Municipality | Act (Legislation) | Chandragiri Health Act, Act No. 8 of the Year 2075 Local Health Service Act, 2075 | 2019 |
| 3 | Bhimeshwor Municipality | Report | Annual Progress Report FY 2080 2081 (2024) | 2024 |
| 4 | Bhaktapur Municipality | Policy | Bhaktapur Municipality Policy and Program of 2080/81 (2024) | 2024 |
| 5 | Bagmati Province, Ministry of Health | Fact sheet | BAGAMATI PROVINCE HEALTH IN FACTS AND FIGURES (2080/81) | 2025 |
|  | **Donor/International development and implementation partner documents** | | | |
| 1 | UNICEF | Terms of reference | TERMS OF REFERENCE (TOR) OF Institutional consultancy to scale up Family MUAC Program in two districts of Nepal | 2024 |
| 2 | UNICEF | Website article | The challenging journey to reach Nepal's communities withvaccines | 2022 |
| 3 | UNICEF | Website article | Smriti Kamar - The first point of contact for commun ities | 2022 |
| 4 | UNICEF Nepal | Report | Nepal: HEALTH - Thematic report. January - December 2018 | 2019 |
| 5 | UNICEF/AUSAid | Report (Evaluation) | Independent Review of Two AusAID Funded UNICEF Projects on Child Survival and Nutrition and Maternal Health in Nepal | 2013 |
| 6 | UNICEF Regional Office for South Asia (ROSA) | Policy brief | Policy Brief for Nepal: Evaluation of South Asia’s Current Community Health Worker Policies and System Support and their Readiness for Community Health Workers’ Expanding Roles and Responsibilities within Post-Astana National Health Care Strengthening Plans | 2022 |
| 7 | UNICEF Regional Office for South Asia (ROSA) | Policy brief | Regional Brief: Evaluation of South Asia’s Current Community Health Worker Policies and System Support and their Readiness for Community Health Workers’ Expanding Roles and Responsibilities within Post-Astana National Health Care Strengthening Plans | 2022 |
| 8 | UNICEF Regional Office for South Asia (ROSA) | Report | Gender Analysis report Evaluation of South Asia’s Current Community Health Worker Policies and System Support and their Readiness for Community Health Workers’ Expanding Roles and Responsibilities within Post-Astana National Health Care Strengthening Plans | 2022 |
| 9 | Nepal Health Research Council (NHRC) / UNICEF | Report | Factors affecting Health-seeking Behaviour among people in Nepal: Exploratory study on Institutional delivery, Routine Child Immunization and COVID-19 vaccination | 2023 |
| 10 | UNICEF / Nepal government Ministry of Finance | Strategic plan | COUNTRY PROGRAMME ACTION PLAN 2018-2022 | 2018 |
| 11 | UNICEF Nepal / Nepal government | Strategic plan | Country Programme Action Plan 2023-2027 | 2023 |
| 12 | UNICEF Nepal / Nepal public health foundation | Evaluation summary | ANM PROGRAMME ASSESSMENT, DADELDHURA, NEPAL (OCT 2017 – MAY 2018) | 2018 |
| 13 | UNICEF Nepal | Website article (blog) | KOICA and UNICEF Representatives visit Baitadi and Dadeldhura | 2015 |
| 14 | Behavioral Science Center (KUSMS), JSI, UNICEF | Report | SUMMARY REPORT: Understanding the Behavioural and Social Drivers for Under-vaccination of Children in Nepal - Findings from Bagmati, Madesh, and Sudurpaschim Provinces | 2023 |
| 15 | Save the Children | Brief | Are Female Community Health Volunteers (FCHVs) Overloaded? | 2016 |
| 16 | Save the Children | Brief | Are Female Community Health Volunteers Dissatisfied? | 2016 |
| 17 | Save the Children | Brief | How do Female Community Health Volunteers Report Spending Their Time? | 2016 |
| 18 | Save the Children | Brief | How Does the FCHV Contribution to MoH Programs Now Compare to the Past? | 2016 |
| 19 | Save the Children | Report (Case study) | CASE STUDY: NEPAL’S EXPERIENCE WITH TREATMENT OF POSSIBLE SEVERE BACTERIAL INFECTION IN NEWBORNS WHERE REFERRAL IS NOT POSSIBLE (2017) | 2017 |
| 20 | USAID | (Technical) Brief | Nepal Family Health Program II (NFHPII) Technical brief #1. "Female community health volunteers". | 2012 |
| 21 | USAID | Report | MCHIP Nepal Annual Report: October 2009–September 2010 (Jhpiego, 2010) | 2010 |
| 22 | USAID | (Technical) Brief | Nepal Family Health Program (NFHP) Technical brief #20 Surveys & Studies | 2007 |
| 23 | USAID | Report | Nepal Family Health Program (NFHP) II Evaluation | 2011 |
| 24 | USAID | Report | Nepal Family Health Program Year Four Assessment | 2005 |
| 25 | USAID / Save the Children | (Technical) Brief | Health and Family Planning Program: SUAAHARA Project – Good Nutrition | 2013 |
| 26 | USAID / HKI | Report | Suaahara II GOOD NUTRITION PROGRAM - SIXTH ANNUAL REPORT. JULY 16, 2021–July 15, 2022 | 2022 |
| 27 | USAID / HKI | (Program) Brief | Applying a Comprehensive SBCC Approach to Improve Maternal and Child Diets: Lessons from Suaahara II | 2023 |
| 28 | USAID / HKI | Brief | Amplifying Local Voices for Inclusive Nutrition Programming | 2023 |
| 29 | USAID / HKI | (Technical) Brief | SUAAHARA II: ADOLESCENT NUTRITION: TECHNICAL BRIEF | 2023 |
| 30 | USAID / HKI | Brief | Onsite coaching as a mechanism to enhance the capacity of healthcare workers | 2023 |
| 31 | USAID / HKI | Brief | Integration of Family Planning in a Nutrition Program: Lessons from USAID’s Suaahara II Program | 2023 |
| 32 | USAID / HKI | Brief | Engaging Private Sector to Improve Access to Nutritious Food and Hygiene Products: Learnings from Suaahara II | 2023 |
| 33 | USAID / HKI | Brief | Engaging Health Facility Staff in Applying National Standards Contributes to Improving WASH Standards | 2023 |
| 34 | USAID / HKI | (Technical) Brief | SUAAHARA II: WATER, SANITATION AND HYGIENE: TECHNICAL BRIEF | 2023 |
| 35 | USAID / HKI | Brief | Promoting Radio Listenership through Localization of Production: A Case Study of Bhanchhin Aama | 2023 |
| 36 | USAID / HKI | (Technical) Brief | SUAAHARA II: HEALTH AND NUTRITION SERVICES: TECHNICAL BRIEF | 2023 |
| 37 | USAID / HKI | Brief | Improving egg consumption in mothers and young children through backyard poultry farming | 2023 |
| 38 | USAID / HKI | (Technical) Brief | SUAAHARA II: GENDER EQUALITY AND SOCIAL INCLUSION: TECHNICAL BRIEF | 2023 |
| 39 | USAID / HKI | Brief | Engaging Fathers to ChangeGender Norms for Improved Health and Nutrition Practices | 2023 |
| 40 | USAID / HKI | Brief | What are the barriers in the Treatment of Severe Acute Malnutrition in Children and how can we overcome them? | 2023 |
| 41 | USAID / HKI | Brief | Empowering communities to work hand-in-hand with their local health staff to improve services | 2023 |
| 42 | USAID / HKI | (Technical) Brief | SUAAHARA II: FAMILY PLANNING: TECHNICAL BRIEF | 2023 |
| 43 | USAID / HKI | (Technical) Brief | SUAAHARA II: GOVERNANCE TECHNICAL BRIEF | 2023 |
| 44 | USAID / HKI | (Technical) Brief | SUAAHARA II: MONITORING, EVALUATION AND RESEARCH: TECHNICAL BRIEF | 2023 |
| 45 | USAID / HKI | (Technical) Brief | SUAAHARA II: NUTRITION SENSITIVE AGRICULTURE: TECHNICAL BRIEF | 2023 |
| 46 | USAID / HKI | Brief | Prevention and Treatment of Wasting among Children: A Call for Action | 2023 |
| 47 | USAID / HKI | Brief | Promoting and Sustaining Homestead Food Production in Marginalized Communities of Nepal Through Village Model Farmers: Lessons from Suaahara II Good Nutrition Program | 2023 |
| 48 | USAID / HKI | (Technical) Brief | SUAAHARA II: SOCIAL AND BEHAVIOR CHANGE (SBC): TECHNICAL BRIEF | 2023 |
| 49 | USAID / HKI | Brief | Suaahara: Strengthening Local Systems to Deliver Nutrition and Health Services in Nepal | 2023 |
| 50 | USAID / HKI | Brief | Suaahara: Evaluating a Decade of Multisectoral Nutrition Interventions in Nepal | 2023 |
| 51 | USAID / HKI | Brief | The SATH Approach: A Cornerstone in Increasing Demand for Health and Nutrition Services among Marginalized Communities | 2023 |
| 52 | USAID / HKI | Booklet | SEVEN YEARS OF USAID’S SUAAHARA II: STORIES OF HOPE AND RESILIENCE | 2023 |
| 53 | USAID Nepal / Save the Children | Report | IMPROVING NUTRITION IN NEPAL: THE SUAAHARA EXPERIENCE | 2016 |
| 54 | UNDP / Ministry of Local Development / Institute of Local Governance Studies |  | Assessment of Village Development Committee Governance and the use of Block Grants | 2009 |
| 55 | WHO | Website | Nepal Successfully Completes Mass Drug Administration Campaign against Lymphatic Filariasis in 15 Districts | 2023 |
| 56 | WHO Regional office for South-East Asia | Brief | Health financing profile 2017 Nepal | 2017 |
| 57 | WHO Nepal / MoHP | Strategy | WHO Country Cooperation Strategy (CCS) 2023–2027 | 2023 |
| 58 | WHO Nepal / MoHP | Strategy | WHO Country Cooperation Strategy Nepal 2013-2017 (2013) | 2013 |
| 59 | WHO Nepal / MoHP | Strategy | Nepal–WHO Country Cooperation Strategy (CCS) 2018–2022 | 2018 |
| 60 | WHO Nepal / MoHP | Strategy | WHO Country Cooperation Strategy Nepal 2006-2011 (2007) | 2007 |
| 61 | Nepal Health Sector Support Programme (UK Aid) / Ministry of Health and Population / BEK | (Technical) Brief | Nepal Health Sector Support Programme - Assessment of Health Facility Operations and Management Committees (HFOMCs) in Nepal | 2023 |
| 62 | Nepal Health Sector Support Programme (UK Aid) / Ministry of Health and Population | Report | Gender Equality and Social Inclusion: From Strategy to Implementation - GESI reflected in Family Health Division and Child Health Division Planning | 2013 |
| 63 | Nepal Health Sector Support Programme (UKAid) / MoHP | Report | Knowledge Café/ Policy Dialogue Proceedings Report: PRIORITISING AND SETTING HEALTH GOALS FOR PROVINCIAL AND LOCAL LEVEL PLANS IN LUMBINI PROVINCE (Nepal Health Sector Support Programme (NHSSP 3) – No Cost Extension) | 2023 |
|  | **International Non-governmental organizations (INGOs)** | | | |
| 1 | ORC Macro / USAID Nepal | Report | Nepal Family Health Program Baseline Assessment Report | 2002 |
| 2 | ORC Macro / USAID Nepal | Report | Nepal Family Health Program Year Two Assessment | 2003 |
| 3 | ORC Macro / USAID Nepal | Report | Nepal Family Health Program Year Three Assessment | 2004 |
| 4 | ILO | (Country) Brief | Social Protection in Action: Building social protection floors for all - Country Brief: Nepal | 2021 |
| 5 | ILO | Report | Social health protection in Nepal: State of play and recommendations towards universal extension of coverage | 2023 |
| 6 | ADB | Report | A STUDY ON NEPAL’S NATIONAL HEALTH INSURANCE PROGRAM | 2024 |
| 7 | JSI | Presentation | Female Community Health Volunteer (FCHV) National Survey 2014 Nepal | 2014 |
| 8 | HERD International / ReBUILD consortium | Website | Health Facility Operation and Management Committees and theirsignificance in local health governance in Nepal | 2024 |
| 9 | BBC Media Action | Report | Female Community Health Volunteers Engagement With Communities And The Feasibility Of Using Mobile Phones As A Job Aid: A Qualitative Study | 2019 |
| 10 | Exemplars in Global Health | Case Study (Report) | UNDER-FIVE MORTALITY REDUCTION IN NEPAL | 2020 |
| 11 | Exemplars in Global Health | Case Study (Report) | Stunting Reduction in Nepal | 2019 |
| 12 | Clean Cooking Alliance | (Policy) Brief | Collaborating with Female Community Health Volunteers in Support of Clean Cooking Programs in Nepal | 2021 |
| 13 | Global delivery intiative | Case Study (Report) | Piloting Innovations in Nepal’s Community Health Worker Program. | 2019 |
|  | **Global trade union federations supporting FCHV unions** | | | |
| 1 | UNI Global Union | Website (News) | NEPAL’S FEMALE COMMUNITY HEALTH WORKERS HONOUREDWITH SPECIAL FINANCIAL AWARD, PRESSES ON FOR FULLEMPLOYMENT RECOGNITION | 2024 |
| 2 | UNI Global Union | Website (News) | VICTORY FOR NEPALESE UNION WINS LANDMARK FREE HEALTHINSURANCE FOR COMMUNITY HEALTH WORKERS | 2023 |
| 3 | Public Services International (PSI) | Website (News) | Union Win: Kathmandu Health Volunteers Will be Recognized As Community Health Workers | 2024 |
| 4 | PSI | Website (News) | Nepal's Trade Unions call to Tighten Corporate Tax Compliance and Wealth Tax on the Rich | 2024 |
| 5 | PSI | Website (News) | Community Health Workers’ submit Charter of Demands to Nepal’s Prime Minister | 2024 |
| 6 | PSI | Website (News) | Empowering Nepal’s Community Health Workers | 2024 |
| 7 | PSI | Website (News) | Community Health Workers from Nepal head to Geneva to demand Rights and Respect | 2024 |
| 8 | PSI | Website (News) | Nepalese Women Unionists to demand Community Health Work is recognised as work at International Labour Conference in Geneva | 2024 |
| 9 | PSI | Website (News) | PSI congratulates CHWs of Bansgadi in Nepal for inclusion in social security scheme | 2024 |
| 10 | PSI | Website (News) | Video Launch and Panel Discussion on Challenges and Triumphs of CHWs in Nepal | 2024 |
| 11 | PSI | Website (News) | Community Health Work is Work! | 2023 |
| 12 | PSI | Website (News) | PSI builds organising capacity of Nepalese CHWs | 2022 |
| 13 | PSI | Website (News) | "Community health work is work" says CHWs in South Asia | 2022 |
| 14 | PSI | Website (News) | Female Community Health Workers in Nepal talk about labor exploitation | 2022 |
| 15 | PSI | Website (News) | #IWD2022 Messages from Community Health Workers | 2022 |
| 16 | PSI | Presentation | Community Health Workers of South Asia Campaign | 2022 |
| 17 | PSI | Briefing Note | Submission to ILO General Survey 2021 on the Nursing Personnel Convention, 1977 (No. 149) and the Nursing Personnel Recommendation, 1977 (No. 157) From Community Health Workers in India, Nepal, Pakistan, Philippines, Malawi, South Africa and Zambia | 2021 |
| 18 | PSI | Briefing Note | COMMUNITY HEALTH WORK IS WORK (Submission to International Labor Conference 2024) | 2024 |
|  | **Non-governmental organizations (NGOs), Nepal-based** | | | |
| 1 | New ERA / MOHP / USAID | Report | An Analytical Report on Female Community Health Volunteers of Selected Districts of Nepal | 2008 |
| 2 | New ERA / MOHP / USAID | Report | Nepal Family Health Program Year Four Assessment | 2005 |
| 3 | New ERA / USAID / ORC Macro | Report | An analytical report on FCHVs in Nepal (2005 survey) | 2006 |
| 4 | New ERA / USAID / MoHP | Report | An Analytical Report on National Survey of Female Community Health Volunteers of Nepal | 2006 |
| 5 | Public Policy Pathshala / The Asia Foundation / AusAID | Report | LOCAL HEALTH GOVERNANCE: SITUATIONAL AND POLITICAL ECONOMY ANALYSIS REPORT | 2020 |
| 6 | Center for Social Change | Policy Brief | Health Governance in Nepal: Based on One Year of Real-Time Governance Monitoring | 2022 |
| 7 | Nyaya Health Nepal | Report | Nyaya Health Nepal Annual Report 2011 | 2011 |
| 8 | Nyaya Health Nepal | Report | Nyaya Health Nepal Annual Report 2012 | 2012 |
| 9 | Nyaya Health Nepal | Report | Nyaya Health Nepal Annual Report 2013 | 2013 |
| 10 | Nyaya Health Nepal | Report | Nyaya Health Nepal Annual Report 2016-2017 | 2017 |
| 11 | Nyaya Health Nepal | Report | Nyaya Health Nepal Annual Report 2017-2018 | 2018 |
| 12 | Nyaya Health Nepal | Report | Nyaya Health Nepal Annual Report 2018-2019 | 2019 |
| 13 | Nyaya Health Nepal | Report | Nyaya Health Nepal Annual Report 2019-2020 | 2020 |
| 14 | Nyaya Health Nepal | Report | Nyaya Health Nepal Annual Report 2022-2021 | 2021 |
| 15 | Nyaya Health Nepal | Report | Nyaya Health Nepal Annual Report 2021-2022 | 2022 |
| 16 | Nyaya Health Nepal | Report | Nyaya Health Nepal Annual Report 2022-2023 | 2023 |
| 17 | Nyaya Health Nepal | Report | Nyaya Health Nepal Annual Report 2023-2024 | 2024 |
| 18 | Possible health | Report | Annual Impact Report 2014 | 2014 |
| 19 | Possible health | Report | Annual Impact Report 2015 | 2015 |
| 20 | Possible health | Report | Annual Impact Report 2016 | 2016 |
| 21 | Possible health | Report | Annual Impact Report 2017 | 2017 |
| 22 | Possible health | Report | Annual Impact Report 2018 | 2018 |
| 23 | Possible health | Report | Annual Impact Report 2019 | 2019 |
| 24 | Possible health | Report | Annual Impact Report 2020 | 2020 |
| 25 | Possible health | Report | Annual Impact Report 2021 | 2021 |
| 26 | Possible health | Report | Annual Impact Report 2022 | 2022 |
| 27 | Possible health | Report | Annual Impact Report 2023 | 2023 |
|  | **Newspaper articles** | | | |
| 1 | Kathmandu Post | News article (web) | Nepal witnesses unprecedented rise in suicide cases (August 19, 2024) | 2024 |
| 2 | Kathmandu Post | News article (web) | Apathy to take preventive measures blamed for an exponential rise in dengue cases (August 20, 2024) | 2024 |
| 3 | Kathmandu Post | News article (web) | Valley polio drive hits 104% coverage, but experts warn of gaps (July 31, 2024) | 2024 |
| 4 | Kathmandu Post | News article (web) | 2.8 million girls aged 10-19 years to be given iron-folic acid (July 17, 2024) | 2024 |
| 5 | Kathmandu Post | News article (web) | KMC intensifies dengue mosquito eradication efforts (June 29, 2024) | 2024 |
| 6 | Kathmandu Post | News article (web) | Malnutrition chronic in remote Makawanpur villages (June 26, 2024) | 2024 |
| 7 | Kathmandu Post | News article (web) | People in 7 districts to be given anti-elephantiasis medicine (April 27, 2024) | 2024 |
| 8 | Kathmandu Post | News article (web) | Experts urge learning to cope with mental health problems the right way (March 17, 2024) | 2024 |
| 9 | Kathmandu Post | News article (web) | Insured will have to bear some of the treatment cost from January 15 (December 28, 2023) | 2023 |
| 10 | Kathmandu Post | News article (web) | Notes on a city’s success story (December 13, 2023) | 2023 |
| 11 | Kathmandu Post | News article (web) | Female community health volunteers are unsung heroes ( December 6, 2023) | 2023 |
| 12 | Kathmandu Post | News article (web) | Identifying mental health patients through female health volunteers (October 7, 2023) | 2023 |
| 13 | Kathmandu Post | News article (web) | Local governments seek male participation to enhance safe motherhood initiatives ( June 7, 2023) | 2023 |
| 14 | Kathmandu Post | News article (web) | More children in cities miss routine vaccinations (May 11, 2023) | 2023 |
| 15 | Kathmandu Post | News article (web) | Anti-elephantiasis drive in 15 districts (February 24, 2023) | 2023 |
| 16 | Kathmandu Post | News article (web) | A volunteer’s act earns praise but exposes health inequities in Nepal (February 4, 2022) | 2022 |
| 17 | Kathmandu Post | News article (web) | When will people get booster shots? (February 1, 2022) | 2022 |
| 18 | Kathmandu Post | News article (web) | Female community health volunteers feel neglected (January 2, 2022) | 2022 |
| 19 | Kathmandu Post | News article (web) | Nepal's vaccination drive against Covid-19 begins (January 27, 2021) | 2021 |
| 20 | Kathmandu Post | News article (web) | Nepal launches its Covid-19 vaccination drive starting with frontline and healthcare workers ( January 27, 2021) | 2021 |
| 21 | Kathmandu Post | News article (web) | Local health workers, doctors to be trained to deal with mental health issues (December 15, 2020) | 2020 |
| 22 | Kathmandu Post | News article (web) | Volunteers, teachers and college students to be trained for contact tracing (July 6, 2020) | 2020 |
| 23 | Kathmandu Post | News article (web) | Female community health volunteers: unsung heroes shouldering huge responsibilities with minimal return (December 6, 2019) | 2019 |
| 24 | Kathmandu Post | News article (web) | Adolescents to be included in mothers’ health groups (December 7, 2019) | 2019 |
| 25 | Kathmandu Post | News article (web) | Health Ministry to distribute misoprostol to prevent postpartum haemorrhage ( October 22, 2019) | 2019 |
| 26 | Kathmandu Post | News article (web) | Women are suffering due to a lack of doctors at district hospitals (September 23, 2019) | 2019 |
| 27 | Kathmandu Post | News article (web) | Female community health volunteers in Baitadi fret over ‘paltry’ remuneration (August 26, 2019) | 2019 |
| 28 | Kathmandu Post | News article (web) | Health budget increased marginally, but experts say it’s insufficient (May 30, 2019 ) | 2019 |
| 29 | Kathmandu Post | News article (web) | City dwellers do not support government health programmes and services (April 22, 2019) | 2019 |
| 30 | Kathmandu Post | News article (web) | ‘FCHVs can play crucial role in fighting NCDs’ (December 12, 2017) | 2017 |
| 31 | Kathmandu Post | News article (web) | Ministry of Health pilots mHealth, eHealth services (July 4, 2017) | 2017 |
| 32 | Kathmandu Post | News article (web) | Female health volunteers want allowances increased (July 24, 2014) | 2014 |
| 33 | Kathmandu Post | News article (web) | Community health worker injured in assault (November 27, 2014) | 2014 |
| 34 | Kathmandu Post | News article (web) | FCHVs seek recognition of their contributions (Dec 9, 2012) | 2012 |
| 35 | Kathmandu Post | News article (web) | Nepal faces health crisis as budget cuts threaten vital services (April 8, 2025) | 2025 |
| 36 | Kathmandu Post | News article (web) | Graduation from LDC and SDG targets under threat (March 31, 2025) | 2025 |
| 37 | Kathmandu Post | News article (web) | Health ministry urges UN bodies for budgetary support amid funding uncertainty (March 20, 2025) | 2025 |
| 38 | Kathmandu Post | News article (web) | Health ministry mulls virtual meetings, training as US aid freeze saps resources (March 14, 2025) | 2025 |
| 39 | Kathmandu Post | News article (web) | In Nepal, US pulls the plug on aid projects worth Rs46.12 billion (March 12, 2025) | 2025 |
| 40 | Kathmandu Post | News article (web) | Health ministry braces for Rs3 billion budget cut next year (March 8, 2025) | 2025 |
| 41 | Kathmandu Post | News article (web) | US aid suspension under Trump disrupts key health surveys in Nepal (February 28, 2025) | 2025 |
| 42 | Kathmandu Post | News article (web) | Nepal to focus on crucial health programmes, seeks donor support (February 19, 2025 ) | 2025 |
| 43 | Kathmandu Post | News article (web) | USAID funding freeze: Ministry terminates jobs of 36 staff nurses in federal, provincial hospitals (February 17, 2025) | 2025 |
| 44 | Kathmandu Post | News article (web) | US cancels $39 million in aid for Nepal’s federalism and biodiversity projects (February 16, 2025) | 2025 |
| 45 | Kathmandu Post | News article (web) | USAID fiasco and the NGO ecosystem (February 16, 2025) | 2025 |
| 46 | Kathmandu Post | News article (web) | Foreign aid cut affects Nepal’s maternal and child health programmes: Minister Paudel (May 19, 2025 ) | 2025 |
| 47 | Himalaya Times | News article (web) | Bicycles distributed to 34 women health workers (Jul 28, 2018) | 2018 |
| 48 | Himalaya Times | News article (web) | EDITORIAL: Forests up a tree (Dec 07, 2018) | 2018 |
| 49 | Himalaya Times | News article (web) | Over 250,000 health workers needed ( Jul 05, 2023) | 2023 |
| 50 | Himalaya Times | News article (web) | Master training of trainers on Child and Adolescent Mental Health concludes in Chitwan (Mar 05, 2021) | 2021 |
| 51 | Himalaya Times | News article (web) | Response to COVID-19 Crisis in Nepal (Apr 01, 2020) | 2020 |
| 52 | Himalaya Times | News article (web) | BPKIHS briefs female health volunteers on obstetric fistula (Jun 02, 2018) | 2018 |
| 53 | Himalaya Times | News article (web) | Rapid COVID-19 antigen test campaign begins in Mustang (May 22, 2021) | 2021 |
| 54 | Himalaya Times | News article (web) | UNICEF lauds Nepal for significant progress in nutrition of mothers, children at risk ( Jan 21, 2022) | 2022 |
| 55 | Himalaya Times | News article (web) | ADB approves $165m loan for COVID-19 vaccines in the country (Jul 23, 2021) | 2021 |
| 56 | Himalaya Times | News article (web) | People above 65 to receive vaccines throughout Nepal from today (Mar 07, 2021) | 2021 |
| 57 | Himalaya Times | News article (web) | EDITORIAL: Vaccines arrive ( Jan 22, 2021) | 2021 |
| 58 | Himalaya Times | News article (web) | EU, UNICEF to prevent malnutrition in Nepal (Jul 03, 2020) | 2020 |
| 59 | Himalaya Times | News article (web) | Lack of trust, awareness limits access to health services (Jun 22, 2019) | 2019 |
| 60 | Himalaya Times | News article (web) | Rs 68 billion allocated for health, population (May 30, 2019) | 2019 |
| 61 | Himalaya Times | News article (web) | Budget of 1.53 trillion unveiled for fiscal year 2019/20 (May 29, 2019) | 2019 |
| 62 | Himalaya Times | News article (web) | Nepal Democratic Forum to prioritise women candidates for local level elections (Apr 17, 2017) | 2017 |
| 63 | Himalaya Times | News article (web) | Five female community health volunteers honoured ( Dec 06, 2016) | 2016 |
| 64 | Himalaya Times | News article (web) | Medical team sent to dengue-hit Chitwan for study ( Oct 06, 2016) | 2016 |
| 65 | Himalaya Times | News article (web) | EDCD in bid to check spread of cholera (Sep 17, 2016) | 2016 |
| 66 | Himalaya Times | News article (web) | President presents govt policy and programme for next fiscal (May 08, 2016) | 2016 |
| 67 | Himalaya Times | News article (web) | FCHVs hailed for their selfless service (Dec 06, 2015) | 2015 |
| 68 | Himalaya Times | News article (web) | Fin Min Mahat presents budget for FY 2015/16 (Jul 14, 2015) | 2015 |
| 69 | Himalaya Times | News article (web) | Govt incentives hope for female health volunteers (Oct 02, 2008) | 2008 |
| 70 | Himalaya Times | News article (web) | Government to fund female health volunteers ( Feb 14, 2008) | 2008 |
| 71 | Himalaya Times | News article (web) | Govt to make NFCHV efficient (Oct 02, 2005) | 2005 |
| 72 | Ekantipur | News article (web) | Demand of health volunteers: We should also be included in social security (5 Dec 2024) | 2024 |
| 73 | Republica | News article (web) | Municipality provides allowances to 70 women health volunteers (June 22, 2021) | 2021 |
| 74 | Nepali times | News article (web) | Enlisting female health volunteers to fight COVID-19 (30 March 2020) | 2020 |
| 75 | Baahrakhari News | News article (web) | प्रदेश र स्थानीय तहमा स्वास्थ्य स्वयंसेविका समिति गठन गर्न मन्त्रालयको निर्देशन (Ministry directs to form health volunteer committees at provincial and local levels) | 2022 |
| 76 | The Rising Nepal | News article (web) | Female health volunteers grieve their contribution unsung (6 December 2023) | 2023 |
| 77 | The Rising Nepal | News article (web) | Free public transportation services for FCHV (18 October 2024) | 2024 |

Abbreviations:

HKI, Helen Keller International. USAID, United States Agency for International Development. UNICEF, United Nations Children's Fund. UNDP, United Nations Development Programme. MoHP, Ministry of Health and Population. ILO, International Labor Organization. ADB, Asian Development Bank. JSI. John Snow Incorporated. AusAID, Australian Agency For International Development. DoHS, Department of Health Services. NSSD, Nursing and Social Security Division. FWD, Family Welfare Division (name after federalisation). FHD, Family Health Division (name until federalisation). MoHP, Ministry of Health and Population.

Footnotes:

Note that some reports are co-published by a central government Ministry and an I/NGO or donor agency, and thus the 'Organization' classification total counts given in the main article may be somewhat misleading.
