# Supplementary material for: Assessment of community health workforce governance in federal Nepal
Source: Health Policy Plan. 2026 Jun 29;41(Suppl 1):i17–37. doi: 10.1093/heapol/czaf088 (PMC13311675; doi:10.1093/heapol/czaf088)
Supplement: czaf088_Supplementary_Data [file czaf088_supplementary_data.zip › Table 1_REV_31.10.25.docx]

**TABLE 1. Analytical CHW governance framework**

| **Dimension** | **Sub-dimension** | **Operationalisation for assessment: Research questions** |
| --- | --- | --- |
| **Inputs:** Institutional structures such as formal and informal rules and division of responsibilities | **Strategic vision** | - Are the expected roles and responsibilities (incl. ownership) of governments and other stakeholders clarified in strategic plans? (1,4,7) - Are strategic goals and priorities for the CHW program formulated in policies/plans? (1-7) |
|  | **Participation & Consensus-orientation** | - Are international donors (development partners), civil society actors, health worker associations (unions), private actors and other stakeholders involved with or represented in decision-making structures and processes? (1,2,3,4,5,6,7) - How are differing interests across stakeholders negotiated and reconciled in decision-making? (1,2,3,6,7) |
| **Processes**: Implementation and execution of rules and responsibilities, incl. administrative procedures and oversight | **Institutional capacity & design** | - Do institutions and individuals have the necessary capacities to implement goals specified in the strategic vision (e.g. financial, management, leadership, human resources, coordination)? (3,4,5,6*,7) |
|  | **Accountability & Transparency** | - How is health system accountability to FCHVs ensured (an enabling environment in terms of supervision, training)? (1,4,7) - How is FCHV accountability to the health system and community ensured (e.g. performance, voicing community concerns)? (3,4,5,7) - How transparent are decision-making processes? (1,4,5,7) |
|  | **Reporting & Information systems** | - What information generation and data reporting mechanisms exist toward monitoring and evaluation? (1,2,3,4,5,6,7) - Is this data made available and utilized in evidence-based planning by decision-makers? (1,4,5) |
|  | **Rule of law &** **Enforcement** | - How are policies, laws and regulations enforced (e.g. penalties for non-compliance)? (1,2,3,5) |
| **Outputs**: Positive aims that health system governance should generate | **Responsiveness** | - How is responsiveness to local health needs ensured (disease burden, competencies)? (1,2,4,5) - How is responsiveness to CHW needs ensured (motivation, satisfaction)? (5) |
|  | **Equity & Equality** | - What policies are in place to ensure CHWs reach underserved/marginalized areas and populations? (1,2) - How is fair treatment of CHWs ensured (e.g. compensation differentials, by ethnicity)? (3, 4) |
|  | **Efficacy & Efficiency** | - Are goals specified in CHW strategic plans achieved efficiently (i.e. without duplication or waste)? (1,2,3,4,5,7)? |

Sources: (1) Siddiqui et al 2009; (2) Lim & Lin, 2001, (3) Dieleman et al., 2011, (4) Kaplan et al., 2013, (5) Sonderegger et al., 2011, (6) Martineau et al., 2022, (7) Chen et al., 2011.

Footnotes : * Martineau et al. (2022) refer to ‘capacity of human resources unit staff’ as ‘a professionalized body of HRH (human resources for health) scientists and planners as well as policy-makers who understand and can support HRH at a strategic level’. They also coin the term ‘health workforce literacy’, referring to the ability to think about terms and conditions of employment, productivity, performance and labor rights. We treat these concepts within the concept of ‘management and leadership capacity’ of individual leaders.

Abbreviations: CHW, Community health worker. FCHV, Female community health volunteer.
