# Supplementary material for: Assessment of community health workforce governance in federal Nepal
Source: Health Policy Plan. 2026 Jun 29;41(Suppl 1):i17–37. doi: 10.1093/heapol/czaf088 (PMC13311675; doi:10.1093/heapol/czaf088)
Supplement: czaf088_Supplementary_Data [file czaf088_supplementary_data.zip › Table 3_REV_31.10.25.docx]

**Table 3. FCHV governance roles for the three government tiers before and after federalism**

| **Gov’t tier** |  | **Before federalism** | **After federalism / changes since 2015 (if any)** |
| --- | --- | --- | --- |
| **Central** | **Ministry of Health and Population** | Determines government incentives, FCHV training curriculum. | Determines federal incentives, training curriculum. Policy formulation, monitoring, coordination. |
|  | **DHS Family Health Div.** *(renamed ‘Family Welfare Div. 2017/18)* | Manage & oversee FCHV program, policy formulation. | Responsibility for some programs in which FCHVs work (e.g. vaccination, family planning, safe motherhood, etc.) |
|  | **DHS Nursing & Social Security Div.** *(2018-)* | *-* | Main regulatory, policy, budgeting, quality, and coordination body for FCHVs. |
|  | **Other DHS div.s** *(e.g. Epidemiology and disease control, Child health)* | Responsible for some programs (e.g. malaria, lymphatic filariasis, HIV, etc.) | (unchanged) |
|  | **Central FCHV Committee** | Policy formulation, review and approval of new programs, implementation support, coordination between programs. Meetings 3 /year. Members: various Ministries and agencies/divisions (13 members), donors/INGOs (7). Chair and Secretary from Family Health Division | *Changes:* Meetings semi-annually.  Members: Ministry and agency/division representatives (18 members), donors (1), NGOs (1). Chair and Secretary from Nursing and Social Security Division; Family Welfare Division represented (1). |
| **Mid** | **Regional health directorate** | Coordination, monitoring, guidance/technical assistance to districts | - |
|  | **Provincial Health Training Centre** | - | Basic and refresher training to FCHVs |
|  | **Provincial FCHV Committee** |  | Policy formulation, review and implement programs, stakeholder coordination, develop training curricula. Semi-annual meetings. Chair and Secretary from Provincial Health Directorate. Other Members: Provincial agencies (8), NGOs (2). |
|  | **Provincial Health Directorate** (under Provincial Ministry of Social Development) |  | Policy formulation, review programs, implementation, coordination, develop (training) curricula. Monitor FCHV incentives |
|  | **Districts** | **District (public) health offices:*** Main FCHV responsibility. Implementation, monitoring, report to regional directorate and Family Health Division. Participate in FCHV Review meetings. Establish & monitor district-level FCHV Fund. | **(District-level) health offices:** Manage logistics, supplies, technical assistance to *palikas*. Collate data from *palikas* on FCHVs, report to Provinces and Nursing and Social Security Division. |
| **Local** | **Local FCHV Committee** *(since 2019)* | - | Review programs & performance. Stakeholder coordination. Formulate procedures and plans, monitor implementation, regulation. Monitor FCHV Fund. Members: *Palika* representatives (3 members), Ward Chairperson (1), FCHV (1), representatives of underserved/marginalized community (2), health-related organisations (1). |
|  | **Local governments** | **Village Development Committee (rural), municipalities (urban):** Operate FCHV Fund. provide uniforms 'from time to time'. Provide recognition to well-performing FCHVs in public venues. Join FCHV Review Meeting if invited. | **Municipalities/*Palikas*:** Primary FCHV responsibility. Implementation, monitoring, and reporting. Draft guidelines. Provide local incentives in coordination with (district) health office; report to Provincial health directorate & Nursing and Social Security Div. Provide FCHV training. Ensure FCHV supply: 1/1,000 people in Terai, 1/600 in hills, 1/150 in mountain regions; financially responsible for additional FCHVs beyond this. Monitor FCHV Fund. |
|  | **Wards** *(lowest administrative structure)* | Members may be invited to FCHV Review meetings. | Determines working area & number of FCHVs based on health facility recommendations. Members may attend FCHV Review meeting; Chairperson is member of local FCHV committee. |
|  | **Local health facilities****** | FCHV activity monitoring (annual reports).  Supervision by AHWs. Supply FCHVs with medicines, materials. Mobilizing FCHVs. Conduct two-day FCHV Review Meetings twice-yearly, attendees: local health workers, local representatives. Collaborate with districts and local government to provide additional support. Support HMGs. | *Changes:* Monthly supervision. Collect FCHV monthly and annual reports. Keep records of FCHV selection and retirement (reported by HMG).  Implementation & stakeholder coordination.  Provide monthly refresher training. |
|  | **Healthy Mother's Group (HMG)** | FCHV selection, replacement (if takes paid job, inactive, retires, death) and retirement approvals. Evaluates FCHVs' work, collects FCHV annual reports, submitting to local health facility. FCHV calls monthly meeting and sets agenda. FCHV acts as Secretary of group. | *Changes:* Evaluate FCHVs' work at least annually, conduct review meeting. Collects FCHV monthly and annual reports. Report on FCHV selection, activities, resignations and replacements, to local health institution. Monitor FCHV Fund. In the event of FCHV vacancy, find replacement within 30 days.  FCHV assists Chairperson in holding monthly meetings. |

**Sources**: (FHD, 2008; FHD, 2010; NSSD, 2019; DHS Annual Reports 2009-2023; Ban *et al.*, 2021).

**Abbreviations**: AHW, auxiliary health worker (incl. Auxiliary health workers and auxiliary nurse midwives). FCHV, female community health volunteer. DHS, Department of Health Services. HIV, human immunodeficiency virus. HMG, healthy mothers’ group. INGO, International non-governmental organization. MOHP, Ministry of Health and Population. Div., division. NGO, non-governmental organization.

**Footnotes**: * Prior to 2015 (federalism), 26 districts had District Public Health Offices and 49 had District Health Offices; despite differing nomenclatures, they performed similar functions. ** ‘Basic Health Service Centres’: Primary Health Care Centers, Health Posts, Community Heath Units and Urban Health Centers. ‘-‘ denotes this governance structure did not exist at this time.
