# Supplementary material for: Assessment of community health workforce governance in federal Nepal
Source: Health Policy Plan. 2026 Jun 29;41(Suppl 1):i17–37. doi: 10.1093/heapol/czaf088 (PMC13311675; doi:10.1093/heapol/czaf088)
Supplement: czaf088_Supplementary_Data [file czaf088_supplementary_data.zip › Table_2_REV_31.10.25.docx]

**Table 2. Informant characteristics**

| **Category** | **Number of informants (%)** |
| --- | --- |
| ***Informant type*** |  |
| Ministry of Health & Population officials | 4 (10%) |
| Local government (*Palika*) policymakers and health administrators | 6 (15%) |
| Public-sector health facility workers (incl. supervisors) | 8 (5%) |
| FCHVs | 6 (15%) |
| FCHV unions | 3 (8%) |
| NGO representatives engaging FCHVs | 4 (10%) |
| International donors & implementation partners | 2 (5%) |
| International and national researchers with longstanding experience evaluating FCHV programs | 7 (18%) |
| *TOTAL* | **40** |
| ***Gender*** |  |
| Male | 15 (37%) |
| Female | 25 (63%) |
| ***Location: Municipality (district)*** |  |
| Bhaktapur (Bhaktapur) | 6 (15%) |
| Bhimeshwor (Dolakha) | 6 (15%) |
| Chandragiri (Kathmandu) | 6 (15%) |
| Lalitpur Metropolitan city (Kathmandu) | 3 (8%) |
| Kathmandu Metropolitan city (Kathmandu) | 18 (%) |
| Virtual | 1 (3%) |

Abbreviations: FCHV, female community health volunteer. NGO, non-governmental organization.
