# Supplementary material for: Assessment of community health workforce governance in federal Nepal
Source: Health Policy Plan. 2026 Jun 29;41(Suppl 1):i17–37. doi: 10.1093/heapol/czaf088 (PMC13311675; doi:10.1093/heapol/czaf088)
Supplement: czaf088_Supplementary_Data [file czaf088_supplementary_data.zip › Table_4_REV_31.10.25.docx]

**Table 4. FCHV Governance opportunities and challenges by actor in federal context**

|  | Opportunities | Challenges |
| --- | --- | --- |
| **Local govts (palikas)** | - Greater autonomy to tailor FCHV programs to local needs - Increased fiscal space for FCHVs/community health - Some coordination with federal govt on drafting policy (e.g. FCHV retirement). | - Some local leaders unfamiliar with FCHVs - Federal govt retain much of ownership, constraining 'decision space’ - Variable internal revenue raising capabilities - Federal FCHV funding as conditional grants - Federal oversight over spending - Low intergovernmental transfers from Provinces - Capacity gaps in leadership and monitoring - Dependent on federal gov. for donor funds - Challenges enforcing FCHV selection, retirement, and political activity, and monitoring performance - Turnover of younger FCHVs |
| **Provinces** | - Main role: FCHV Training Centres - Some role in policy formulation & budgeting (e.g. incentives) | - New entity, unclear role in community health - Reduced authority and human resources at district satellite offices - Leadership capacity shortcomings - Provincial Training Centres understaffed and underfinanced; coordination gaps with *palikas* |
| **Federal Govt (MoHP)** | - Minimum educational requirements enable more literate FCHV cadres (successful in urban settings) - More robust data reporting | - Coordination challenges across disease-specific FCHV programs run by different DoHS divisions, some task duplication - Reporting from *palikas* challenged by uneven health facility access to computers & internet - Enforcement of federal priorities (e.g. minimum education, retirement, political involvement), challenged by poor enforcement by HMGs - Donor funding decreased, expected to decline after 2026 Least Developed Country graduation - USAID withdrawal threatens future of planned national FCHV program evaluation |
| **International donors** | - Continued role in funding, planning, drafting policy - Represented on federal FCHV Committee - Deputed staff at Provinces, discussing direct contracting with *palikas*, Provinces | - Reduced representation on federal FCHV Committee post-federalism (7 seats to 1 seat) - Shift in responsibility from Ministry’s Family Welfare to Nursing Division posed some challenges - Decline in government FCHV funding requests |
| **FCHVs and unions** | - Increased participation in local decision-making, included on *palika* FCHV committee, and local review meetings - Strong community recognition; Some elected into local governments - Unions have some influence on federal and local policy - Federal incentives paid through direct electronic bank transfers - Widened role over time, addressing NCDs and social determinants | - Unions not consistently included in decision-making; no seat on FCHV committee or local review meetings - Unions report accountability fragmentation across three government tiers - Low literacy and high age threatens community legitimacy; gov’t literacy programs ineffective - Pilots to replace in urban areas - Reduced pre-service training (18 to 10 days), minimum education policy challenges enrolling marginalized groups as FCHVs - Feelings of inequity from local incentive variation - Inadequate training, supervision, role clarity - Overburdening |
| **NGOs** | - Represented on federal, provincial & *palika* FCHV committees - Involvement in drafting Community Health guideline, provide technical support to MoHP |  |

*Abbreviations*: FCHV, Female community health volunteer. DoHS, Department of Health Services. HMG, Healthy Mother’s Group. MoHP, Ministry of Health and Population. NCD, non-communicable disease. NGO, Non-governmental organization. USAID, United States Agency for International Development.

Sources: Primary and secondary data.
