# Supplementary figures and images for: Assessment of community health workforce governance in federal Nepal
Source: Health Policy Plan. 2026 Jun 29;41(Suppl 1):i17–37. doi: 10.1093/heapol/czaf088 (PMC13311675; doi:10.1093/heapol/czaf088)

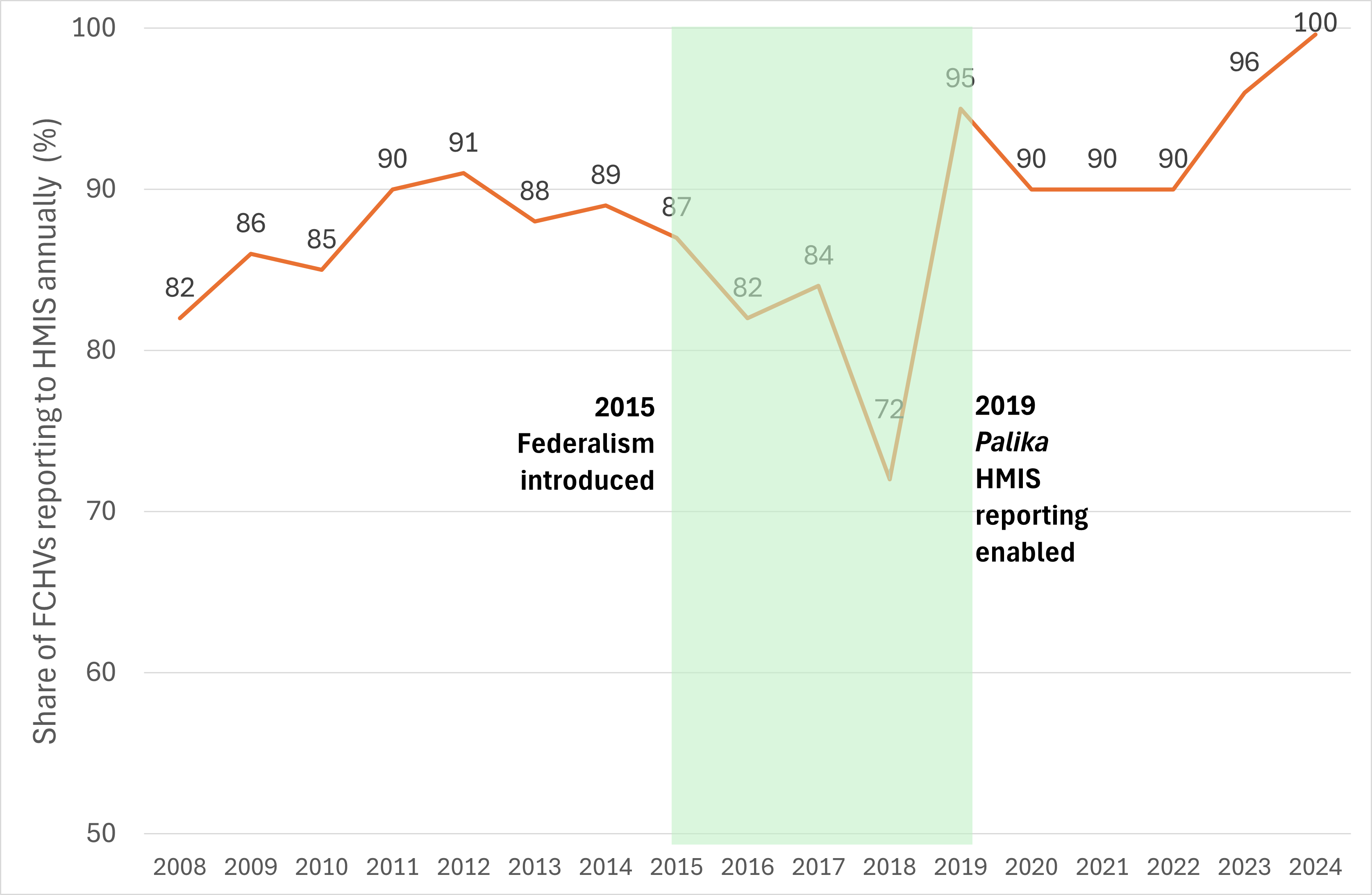

Supplement: czaf088_Supplementary_Data [file czaf088_supplementary_data.zip › Figure 2_Reporting.png]

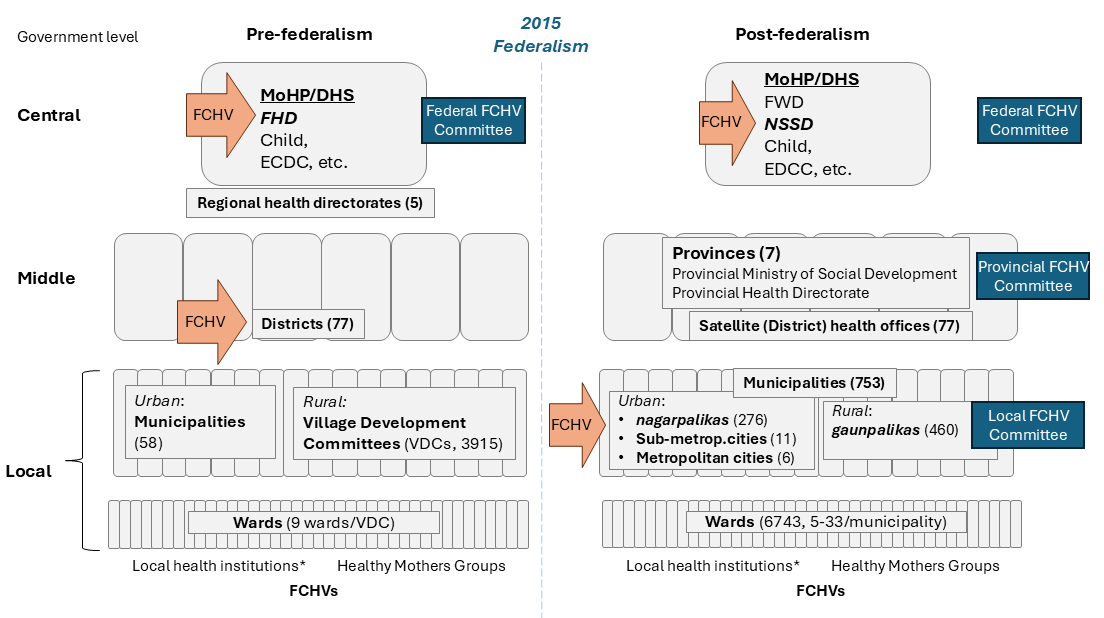

Supplement: czaf088_Supplementary_Data [file czaf088_supplementary_data.zip › Figure1_REV.tif]
